# Fabrication, Functionalization, and Application of Carbon Nanotube-Reinforced Polymer Composite: An Overview

**DOI:** 10.3390/polym13071047

**Published:** 2021-03-26

**Authors:** Norizan Mohd Nurazzi, M.R.M. Asyraf, Abdan Khalina, Norli Abdullah, Fatimah Athiyah Sabaruddin, Siti Hasnah Kamarudin, So’bah Ahmad, Annie Maria Mahat, Chuan Li Lee, H. A. Aisyah, Mohd Nor Faiz Norrrahim, R. A. Ilyas, M. M. Harussani, M. R. Ishak, S. M. Sapuan

**Affiliations:** 1 Institute of Tropical Forestry and Forest Products (INTROP), Universiti Putra Malaysia (UPM), UPM Serdang, Selangor 43400, Malaysia; atiyah88@gmail.com (F.A.S.); chuanli_91@hotmail.com (C.L.L.); a.humaira.aisyah@gmail.com (H.A.A.); mmharussani17@gmail.com (M.M.H.); sapuan@upm.edu.my (S.M.S.); 2 Centre for Defence Foundation Studies, Universiti Pertahanan Nasional Malaysia (UPNM), Kem Perdana Sungai Besi, Kuala Lumpur 57000, Malaysia; 3 Department of Aerospace Engineering, Universiti Putra Malaysia, UPM Serdang, Selangor 43400, Malaysia; mohdridzwan@upm.edu.my; 4 School of Industrial Technology, Universiti Sains Malaysia, Pulau Pinang 11800, Malaysia; 5 School of Industrial Technology, Faculty of Applied Sciences, Universiti Teknologi MARA (UiTM), Shah Alam, Selangor 40450, Malaysia; sitihasnahkam@uitm.edu.my (S.H.K.); sobah@uitm.edu.my (S.A.); 6 Centre for Functional Materials and Nanotechnology, Institute of Science, Universiti Teknologi MARA, Shah Alam, Selangor 40450, Malaysia; anniemaria@uitm.edu.my; 7 Research Center for Chemical Defence, Universiti Pertahanan Nasional Malaysia (UPNM), Kem Perdana, Sungai Besi, Kuala Lumpur 57000, Malaysia; faiznorrrahim@gmail.com; 8 School of Chemical and Energy Engineering, Faculty of Engineering, Universiti Teknologi Malaysia (UTM), Skudai, Johor 81310, Malaysia; ahmadilyas@utm.my

**Keywords:** carbon nanotubes, SWCNT, MWCNT, polymer composites, covalent functionalization, non-covalent functionalization, CNT nanocomposites

## Abstract

A novel class of carbon nanotube (CNT)-based nanomaterials has been surging since 1991 due to their noticeable mechanical and electrical properties, as well as their good electron transport properties. This is evidence that the development of CNT-reinforced polymer composites could contribute in expanding many areas of use, from energy-related devices to structural components. As a promising material with a wide range of applications, their poor solubility in aqueous and organic solvents has hindered the utilizations of CNTs. The current state of research in CNTs—both single-wall carbon nanotubes (SWCNT) and multiwalled carbon nanotube (MWCNT)-reinforced polymer composites—was reviewed in the context of the presently employed covalent and non-covalent functionalization. As such, this overview intends to provide a critical assessment of a surging class of composite materials and unveil the successful development associated with CNT-incorporated polymer composites. The mechanisms related to the mechanical, thermal, and electrical performance of CNT-reinforced polymer composites is also discussed. It is vital to understand how the addition of CNTs in a polymer composite alters the microstructure at the micro- and nano-scale, as well as how these modifications influence overall structural behavior, not only in its as fabricated form but also its functionalization techniques. The technological superiority gained with CNT addition to polymer composites may be advantageous, but scientific values are here to be critically explored for reliable, sustainable, and structural reliability in different industrial needs.

## 1. Introduction 

In 1991, the discovery of carbon nanotubes (CNTs) by Sumio Iijima created a global scientific phenomenon in the field of nanotechnology [1]. A CNT is defined as a one-atom thick sheet of graphite rolled into a tube with a diameter of one nanometer, which is classified as a single-wall carbon nanotube (SWCNT); if there are additional or multiple graphene tubes around the core of an SWCNT, this is known as a multiwalled carbon nanotube (MWCNT). Diameters are fractions of nanometers and tens of nanometers. Lengths can be up to a number of centimeters, with both their ends normally capped by fullerene-like structures [2]. It is believed that the unique properties of CNTs have opened new era in the material world, especially in the field of conductive polymer and CNT-based nanocomposites. Since then, different kinds of techniques of have been developed for CNT-incorporated polymer matrices with the aim to fabricate new advanced materials with multifunctional properties. Some of these properties were designed to transfer the unique electrical properties associated with CNTs to insulating polymer matrices with the aim of obtaining better conducting polymer composites.

Theoretical and experimental results on CNTs have showed a high modulus of elasticity: greater than 1 TPa (the elastic modulus of diamond is 1.2 TPa). In addition, CNTs also possess a strength that is 10–100 times higher than the resilient steel at a fraction of the weight [3]. Additionally, CNTs have an excellent thermal stability of up to 2800 °C in vacuum and an electrical conductivity in the vicinity of 10^3^ S/cm, with an electric-current-carrying capacity that is 1000 times higher and thermal conductivity of about 1900 W m^−1^ K^−1^ (which is about twice as high as diamond) [4,5]. SWCNTs in a hexagonal honeycomb structure consist of sp^2^ hybridized carbon in a that is rolled into a hollow tube morphology, while MWCNTs consist of multiple concentric tubes encircling one another [6]. To date, CNTs have shown increasing interest as potential conductive fillers and reinforcements for polymeric composites. Apart from their high electrical conductivity, CNTs have unique electronic and optical properties for the development of organo-electronic devices [7]. High conductivity can be achieved at a very low concentration of CNTs of between 0.0025 and 4 wt.%, owing to their high aspect ratio (L/D, where L is length of a CNT and D is diameter of a CNT) from hundreds to 1000. All these superiorities allow CNTs to have tremendous potential for nanotechnology fields, especially for use as composite fillers and reinforcements in order to enhance the mechanical, electrical, and thermal properties of resulting composite systems. Many potential applications for CNTs, including microwave absorption [8,9], corrosion protection [10,11], reinforced materials in natural fiber composites [12,13], electromagnetic interference shielding (EMI) [14,15], batteries [16,17], solar cells [18,19,20,21], chemical sensors [22,23,24], hydrogen storage [25,26], field-emission materials [27,28], and adsorbents [29,30], have been reported.

Besides the aforementioned characteristic of CNTs, the rigidity, chemical inertness, and strong π–π interactions of pristine CNTs cannot be synthesized and fabricated due to the difficulties of dissolving or dispersing them in common volatile organic solvents or polymeric matrices. Such actions rely on the agglomeration properties of nanotubes while considering the electrostatic interaction and Van der Walls forces of CNTs that impart the low dispersion properties [31]. Furthermore, the physical nature of the nanosized CNTs plays an important role in dispersing them into a polymer matrix, as well as for a polymer to encapsulate onto a CNT surface. It has been proven that these bundles and agglomerates led to the deterioration of the mechanical and electrical properties of composites compared to the theoretical predictions for individual CNTs [32]. In other words, the dispersion of CNTs does not merely depend on the geometrical problem that related to the length and size of the CNTs alone; instead, it involves a technique that separates individual CNTs from highly entangled and agglomerated CNTs and then stabilize the CNTs in a polymer matrix in order to avoid further agglomeration [33]. Thus, the chemical modification of the side walls of the surfaces of CNTs is needed to improve their dispersion or solubility in solvents or polymers, as well as to improve their interaction and reactivity with polymers by hydrogen bonding interaction [34].

## 2. Synthetization of Carbon Nanotubes

Various techniques have been reported in the literature for the synthesis of CNTs, namely arc discharge, chemical vapor deposition (CVD), the sol–gel process, laser ablation, and electric arc discharge [35]. In the arc discharge technique, direct current is transmitted through graphite electrodes under inert argon at low pressure. The basic concept of laser ablation is similar to arc discharge. The furnace uses a pulsed laser as a point of heat source to provide heat. When using this technique, high-carbon vapor is released from the graphite. The flowing gas is used to move the carbon vapor and the argon. Meanwhile, in CVD, hydrocarbons are used as precursors in the presence of metal catalysts at temperatures varying from 500 to 1000 °C. The hydrocarbons are decomposed at this temperature and, thus, the development of the CNTs as the system cools. The CVD technique is considered the most commonly employed for the mass production of CNTs. Examples of CVD techniques for the production of CNTs include hot-wire (HWCVD), hot-filament (HFCVD), microwave plasma-enhanced (MWCVD), oxygen-assisted, aerosol-assisted (ACVD), and liquid-injection (LICVD) [36]. Table 1 shows the advantages and limitations of some of techniques used in the preparation of CNTs. Being classified as SWCNT and MWCNT, CNTs comprise different chiralities such as arm chair, zig zag, and chiral, all of which affect their structures, properties, and applications [31,37]. Based on reviews, the arm chair chirality has similar properties to metals, while the zig zag and chiral chiralities are more likely to be semiconductors [38]. Additionally, diameter and chiral angle also play important roles in different CNT properties. Therefore, the splendid properties of CNTs may hinder and provide challenges for synthetization techniques.

## 3. Functionalization of Carbon Nanotubes

Modifying the sidewalls of CNTs may affect the solubility characteristic, which can affect the fabrication properties of CNT nanocomposites [39]. Zhao and Stoddard (2009) [40] reported the possibilities of the non-covalent functionalization by small molecules, grafting or wrapping CNTs with polymers in order to improve the electrochemical properties of the material itself. The incorporation of chemically functionalized CNTs into polymers imparts enhancements in mechanical performance because it allows for chemical covalent bonding between CNTs and a polymer matrix. The utilization of chemical functionalization to achieve covalent linkages has been done in SWCNT-reinforced polymer composites [41]. In addition, the use of chemical functionalization is also needed to enhance the nanotube–polymer interface. Such a modification allows for the increases of the interfacial bonding between SWCNTs and the polymer, which leads to improvements in interfacial strength. This improvement enhances the load transfer mechanism of the SWCNTs with the purpose of increasing the macroscopic and microscopic mechanical performance of composite systems [38].

Several methods of functionalization are chemical [42], electrochemical [43], mechano-chemical [44], and plasma [45] in nature. The functionalization of CNTs is used to functionalize their surfaces and side chains. The most common method of chemical functionalization is the one that uses strong acids to remove the end caps and to shorten the length of CNTs. Additionally, oxide groups—particularly carboxylic acids, carbonyl, and hydroxyl groups—are added to the ends and defect sites of CNTs through acid treatments [46]. Further chemical reactions can be performed on these oxide groups to be functionalized with other groups such as amides and thiols [47,48]. Balasubramanian and Burghard (2005) [49] grouped the covalent functionalization of SWCNTs method into three different approaches, namely thermally activated chemistry, electrochemical modification, and photochemical functionalization.

The interfacial bonding between CNTs and polymer matrixes plays a significant role in the properties enhancement of CNT-reinforced polymer composites. Weak interactions between CNTs and polymer matrixes leads to poor interfacial adhesion and thus to CNT agglomeration and aggregation within the polymer matrix. Consequently, the mechanical, thermal, and electrical performance of the composite is weakened [50]. In light of this, the functionalization of CNTs is a potential way to counter problems arising from the fabrication, interfacial adhesion and interaction between matrixes and other reinforcements. The improvement in the interfacial bonding between CNTs and polymer matrixes can help to develop better interfacial strength and thus improve the load transfer mechanism of the CNT structures [51,52].

**Figure 1:**
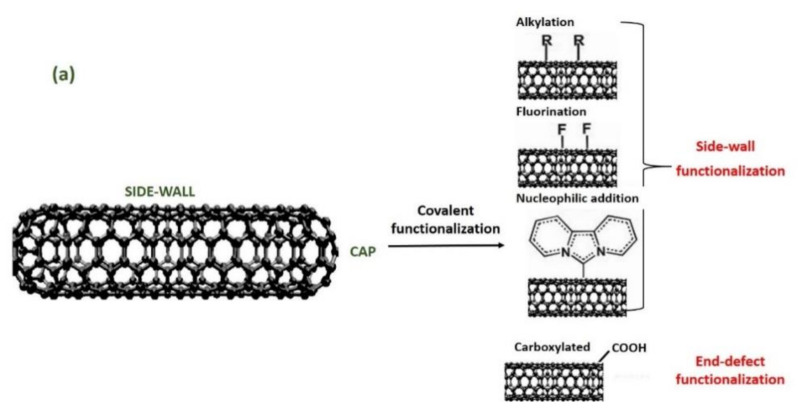

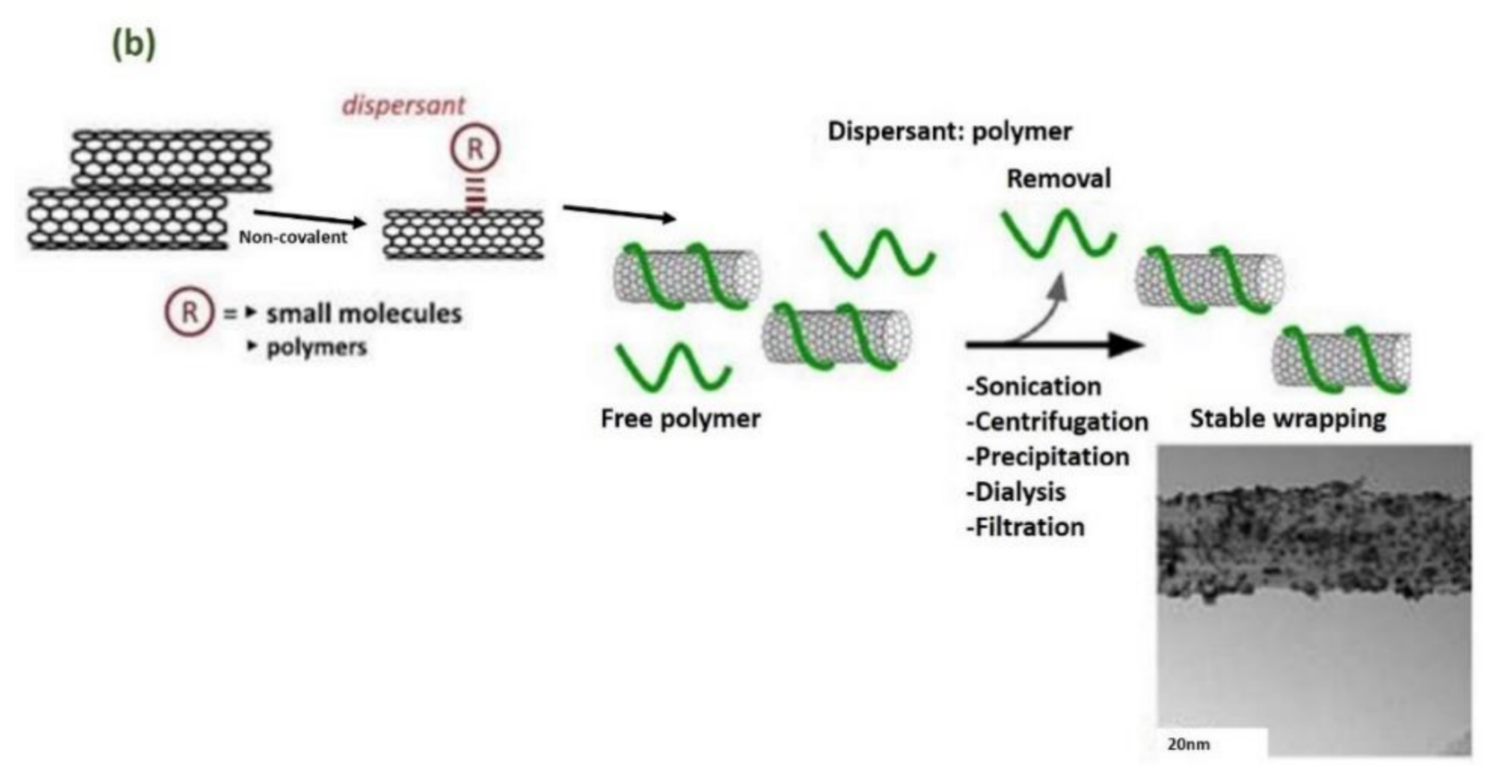
(**a**) The covalent functionalization phenomena at the side and end-caps of CNT structure (reproduced from [33]) and (**b**) non-covalent functionalization method for polymer wrapping (adapted from [54]).

CNTs may be modified to functionalize their side-walls and side chains. Treatments using strong acids to remove the end caps and to shorten the length of the CNTs are considered the most common treatments applied to modified the CNTs and are known as chemical functionalization. Chemical functionalization via acid treatment allows for the addition of oxide groups—mainly carboxylic acids, carbonyl, and hydroxyl groups—to the tube ends and defect sites of CNTs (Figure 1(a)). Extended chemical reactions can be performed on these oxide groups to functionalize them with other functional groups such as amides and thiols [47,48]. Functionalization of CNTs by the attachment of suitable functional groups onto their conjugated sp^2^ carbon scaffold is the requirements for expediting the solubility and ease of dispersion, manipulation, and processibility. In the present section, the current state of functionalization is reviewed, differentiating the covalent and non-covalent functionalization of CNT-reinforced polymer composites (Figure 1) [41,53]. 

### 3.1. Covalent Functionalization

The covalent functionalization of CNTs can be attained by either the direct addition reactions of functional groups to the nanotube sidewalls or through the modification of appropriate surface-bound functional groups on the nanotube ends (Figure 1a) [55,56]. Fundamentally, the method engaged to functionalize CNTs via covalent functionalization is an oxidation process. This reaction allows for the formation of carboxyl groups on the surface of CNTs. However, the structure of a CNT might be affected by the strong oxidizing agent used in covalent functionalization through the changing of a CNT’s structure from a strong sp^2^ hybrid graphite structure to a weak sp^3^ hybrid carbon structure, thus producing a large number of defects on the CNTs. Despite this, oxidation has become a must in functionalization due to it oxidatively introducing carboxyl groups, which is useful for the next functionalization approach. These carboxyl groups help to produce the covalent coupling of molecules through the creation of amide and ester bonds [57]. Ultimately, there are two categories of acid treatments: one refluxes the nanotubes with a solution of nitric acid [58] and the second exposes the sample to a mixture of sulfuric acid/nitric acid (HNO_3_/H2SO_4_) (1:3 by volume) under high power sonication for a maximum of 6 h of reaction time [59].

Consequently, the better dispersion of CNTs in a range of polar solvents, including water, can be achieved via functionalization [60]. Plus, the stacking and layering properties of CNTs can be modified through the covalent attachment of functional groups by altering the hydrogen bonding through reduction of the Van der Waals forces between the CNTs. This strongly enables the de-bundling of CNTs into aligned and individual nanotubes [61]. On the other hand, the covalent functionalization enables the improvement of the inherent properties of the CNTs by changing their electrical conductivity and thermal properties. However, there are few CNTs applications that are suitable for covalent modification, so non-covalent functionalization is preferred. Additionally, the carboxyl groups formed on nanotube surfaces are prone to react in the localized defects of functionalized CNTs and suitable reactive organic groups with other chemical chains. Furthermore, the employment of concentrated acid with the combination of a high power sonication can contribute to the deterioration of CNTs by creating a large number of defects in the sidewalls and, even worse, by fragmenting the CNTs into smaller pieces. These defects can result in the severe deterioration of the mechanical, electrical, and thermal properties of CNTs [31].

Referring to work of Jian and Lau (2020) [62], covalent functionalization is one of the most efficient methods to improve load transfer ability by implementing bonded interactions between the CNTs and polymer matrixes. For example, for epoxy-based nanocomposites, covalent attachment on CNTs via epoxy or amine functional groups is preferred [63].This attachment via amine (NH_2_) functional groups is attributed to the high reactivity with which they can react with an epoxy matrix to form more than one covalent bond during the cross-linking process. This modification of CNTs, therefore, can lead to the improvement of epoxy nanocomposites compared to nanocomposites with unmodified CNTs [64]. Additionally, chemical treatment also helps to obtain a better dispersion of CNTs in an epoxy matrix, because surface functionalization helps to prevent CNTs from agglomerating and forming bundles. The finding of Zhang et al. (2016) [65] showed that surface functional groups affect the dispersion stability of MWCNTs during the mixing and curing process of the epoxy–amine matrix. The study also compared the chemical mechanism of an epoxy matrix containing COOH-MWCNTs that showed re-aggregation during the fabrication process of an epoxy matrix, whereas, the NH_2_-MWCNTs maintained dispersion stability due to the presence of amine-curing agents that ensured that NH_2_-MWCNTs more efficiently participated in the cross-linking reaction with the epoxy matrix.

In a separate study, an acid treatment via a mixture of H_2_SO_4_/HNO_3_ and silane functionalization by 3-aminopropyl triethoxysilane was used to improve the dispersion of MWCNTs with an unsaturated polyester matrix [66]. The results showed that the compressive properties of CNT-modified kenaf composite systems were significantly enhanced via the inclusion of the acid treatment and silane. Meanwhile, silane functionalization presented better improvements of the distribution and bonding between the epoxy matrix and the CNTs, which offset the inferiority of the compressive properties of the kenaf composites. As a result, functionalized CNTs contribute to a better performance of kenaf fiber composites via three main factors: (1) the good dispersion of functionalized CNTs in epoxy, (2) better load transfer mechanisms between the epoxy matrix and the CNTs as fillers due to the formation of covalent bonds, and (3) an improvement of the interfacial adhesion between the epoxy matrix and the CNT surfaces.

### 3.2. Non-Covalent Functionalization

Non-covalent functionalization refers to the adsorption of organic or inorganic molecular chains onto CNTs via several means including π–π stacking, CH–π, hydrogen bonding, and electrostatic interaction. Through this functionalization, the structure of CNTs can remain almost unchanged and the initial mechanical properties of CNTs can be effectively preserved. According to Bose et al. (2010), non-covalent functionalization is an efficient option to shape a CNT and polymer interface, as well as to maintain the reliability of the nanotubes. This method is particularly attractive due to its possibility to absorb various ordered structure groups on CNT surfaces without interfering with the extended π-conjugation of the nanotubes, as well as its ability to create linking with the surface coating/wrapping of low-molecular-weight surfactants (anionic/cationic) [67], polymers [68], liquid crystalline p-conjugated oligomers [69], and amphiphilic cationic polymer molecules [70].

Figure 1b shows a schematic diagram of the non-covalent functionalization paths of polymers towards CNTs [54]. Furthermore, the non-covalent functionalization works via enthalpy-driven interactions between the CNT surfaces and the dispersants and/or entropy-driven interactions, e.g., hydrophobic interaction using surfactants [54]. For surfactant dispersion, sodium dodecyl sulfate [71], sodium dodecyl benzene sulfonate [72], sodium cholate [73], cetyltrimethylammonium bromide [74], Brij, Tween, and Triton X [75] have typically been used due to their availability and low cost [76]. However, non-covalent bonding, such as hydrogen bonding and π–π stacking, is relatively weak in comparison to covalent bonding. The polymer chains or organic molecules attached onto MWCNTs can desorb from MWCNT surfaces when MWCNTs are filtered and re-dispersed or the solvent changes [77].

Several findings on the non-covalent functionalization methods by using functional polymers to wrap onto CNT surfaces have been reported. Results have shown improvements of the mechanical properties of polymer composites [78]. For example, Mandal and Nandl (2012) fabricated nanocomposites using polythiophene (PTh)-wrapped MWCNTs via π–π stacking interactions. After the addition of 0.05 wt.% of functionalized MWCNTs, the Young′s modulus, tensile strength, and fracture toughness of the poly (vinylidene fluoride) (PVDF) composites were two-to-three times higher than those of unfunctionalized MWCNTs. 

Cha performed a different study on the dispersion of CNTs in epoxy matrix composites using polystyrene sulfonate (PSS) and poly(4-aminostyrene) (PAS) that attached to the surfaces of CNTs by non-covalent functionalization. In the case of PAS, the amino group chemically bonded with the epoxide groups in the epoxy groups. The non-functionalized CNTs with PSS and PAS showed enhanced mechanical properties when incorporated into the epoxy nanocomposites. This finding proved that when non-covalent functionalized CNTs were incorporated into a modified bisphenol-A type epoxy matrix, it yielded a Young′s modulus of 3.89 GPa and a tensile strength of 82.59 MPa with the addition of only 1 wt.% PAS-CNTs. The mechanical properties of the nanocomposites were improved due to better dispersion and strong affinity of the epoxy matrix imparted from the noncovalent functionalization [79].

The effect of percolation on the electrical conductivity of non-covalently amino molecule-coated MWCNT/epoxy nanocomposites was investigated by Zhang et al. (2012) [80] by attaching the organic amino molecules of a tetrazine compound onto the surfaces of MWCNTs. The amino groups chemically bonded with epoxide groups in the epoxy of MWNT/epoxy nanocomposites and imparted a higher conductivity compared to that of pristine MWCNT/epoxy nanocomposites at a similar MWCNT content. The percolation threshold of the amino coated MWCNT/epoxy nanocomposites was found to be 0.13 wt.%, while that of pristine MWNT/epoxy composites was 0.51 wt.%. The lower percolation threshold indicated that the effectiveness of amino group attachment onto the surface of MWCNTs via non-covalent functionalization. This led to a better improvement of the dispersion of the MWCNTs in the epoxy matrix, which is important for MWCNTs to form an electrically conductive network to enhance electrical conductivity.

## 4. Fabrication Technique Involved in Carbon Nanotube Polymer Composites

**Figure 2:**
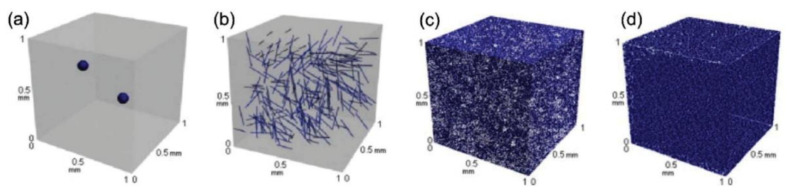
Distribution of micro- and nano-scale fillers: (**a**) Al_2_O_3_ particle, (**b**) carbon fiber, (**c**) graphene nano-platelets (GNPs), and (**d**) CNTs. Adapted from [82].

In practice, there are a variety of techniques to incorporate and fabricate CNTs with a polymer matrix. Several parameters that control the maximization performance of CNTs in polymer composites include the types of fabrication, dispersion, and orientation of CNTs; the length of the CNTs; the matrix; and the chirality. Many attempts have been developed to use CNTs as outstanding nanofillers to strengthen polymer matrixes for CNT-reinforced polymer composite production [81]. The effect of nano-scale CNT distribution leads to an exceptionally large surface area within the nanocomposites compared to other carbon-based micron fillers, as depicted in Figure 2.

### 4.1. Melt Mixing

**Figure 3:**
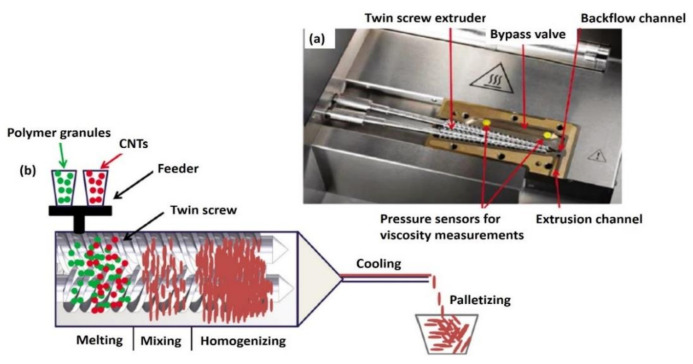
(**a**) View of micro-compounder with different valves and channels; (**b**) the schematic representation of a twin-screw extruder for the melt mixing of CNT-reinforced nanocomposites. Adapted from [81,85].

Melt mixing is an industrial-friendly process for the production of thermoplastic-based nanocomposites. This method was recommended because it is fast, inexpensive, and suitable for insoluble polymers (particularly thermoplastics that cannot be processed in common solvents). Generally, this technique involves the blending of polymer matrixes with CNTs, which are then subject to shear forces at elevated temperatures using a Banbury mixer or an extruder machine that contributes to the disruption and dispersion of CNT bundles in a polymer matrix. Less viscous thermoplastics ease the blending process with nanotube bundles as the temperature increases [81,83,84]. This requires no solvents/chemicals, supports a high volume of bulk polymers, and has a low cost with the least effects on the environment, thus making this technique the best choice to fabricate CNT-based nanocomposites [5]. The process generally involves the blending of polymers with CNTs, usually at high filler contents continuously or in the form of batches at a certain level of temperature when using a extruder or a high shear mixer (Figure 3), respectively. A high shear mixer is often used to prepare master-batches of nanocomposites that are highly concentrated with the desired amount of CNTs loaded in the nanocomposites. However, the use of high shear force and temperature might break the CNTs and polymer chains [81,82].

### 4.2. Solution Mixing

Solution mixing is the most common technique to form CNT-based polymer nanocomposites. This techniques involves intensive agitation (e.g., refluxing, mechanical/magnetic stirring, vigorous shaking, high shear homogenization, and bath/probe sonication) to rigorously aid the mixing of CNTs with polymers in a solvent with the aim to facilitate nanotube de-bundling and their dispersion inside a host polymer matrix [37,86]. This method is able to fabricate nanocomposites without losing the properties of the nanoparticle, as the dispersion and interfacial bonding of the filler are very important to impart good mechanical and electrical properties. Numerous studies on the formation of CNT-based nanocomposites have been carried out with this method (using both organic and aqueous media, as well as a variety of polymer matrices) [37,87,88]. CNTs are dispersed into a suitable solvent by stirring, mixing, or sonication while applying mechanical energy to unbundle the CNTs. The dispersed CNTs are then mixed with a polymer, followed by a controlled evaporation process. Finally, the dispersed CNTs and polymer matrix are mixed and form a composite film [89].

The choice of solvent is very important, as it depends on the solubility of the polymer matrix. The solvents for CNTs and a polymer matrix may be the same or different, but they must have good miscibility. Additionally, the boiling point of the solvent has been found to have a remarkable influence on the properties of formed nanocomposites. Low-boiling point solvents are generally preferred because they are easily removed from the mass of solution-made nanocomposites. On the other hand, high-boiling point solvents are difficult to be removed and tend to get trapped in the solidifying/curing mass. The trapped solvent may obstruct the curing reaction (in thermosets) or can act as a softener (in thermoplastics), thereby deteriorating the electrical, thermal, or mechanical properties [90]. Therefore, surfactants can be applied for tube dispersion or physically/chemically functional CNTs can be used in order to improve dispersion and to solve the problem of tube shortening after high-power agitation [91,92,93]. Nevertheless, solution processing is still broadly used and is one of the key steps in the formation of thermosetting-reinforced nanocomposites.

### 4.3. Sonication

**Figure 4:**
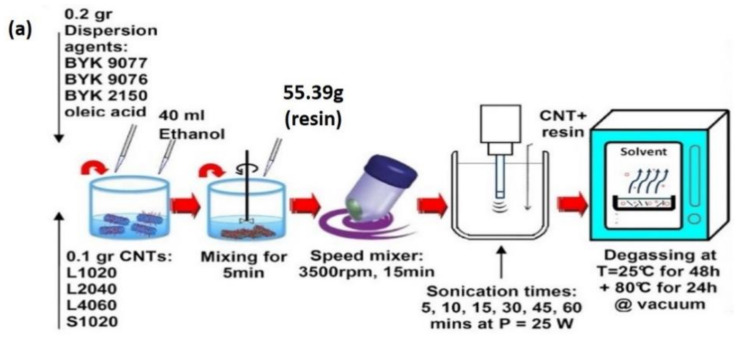

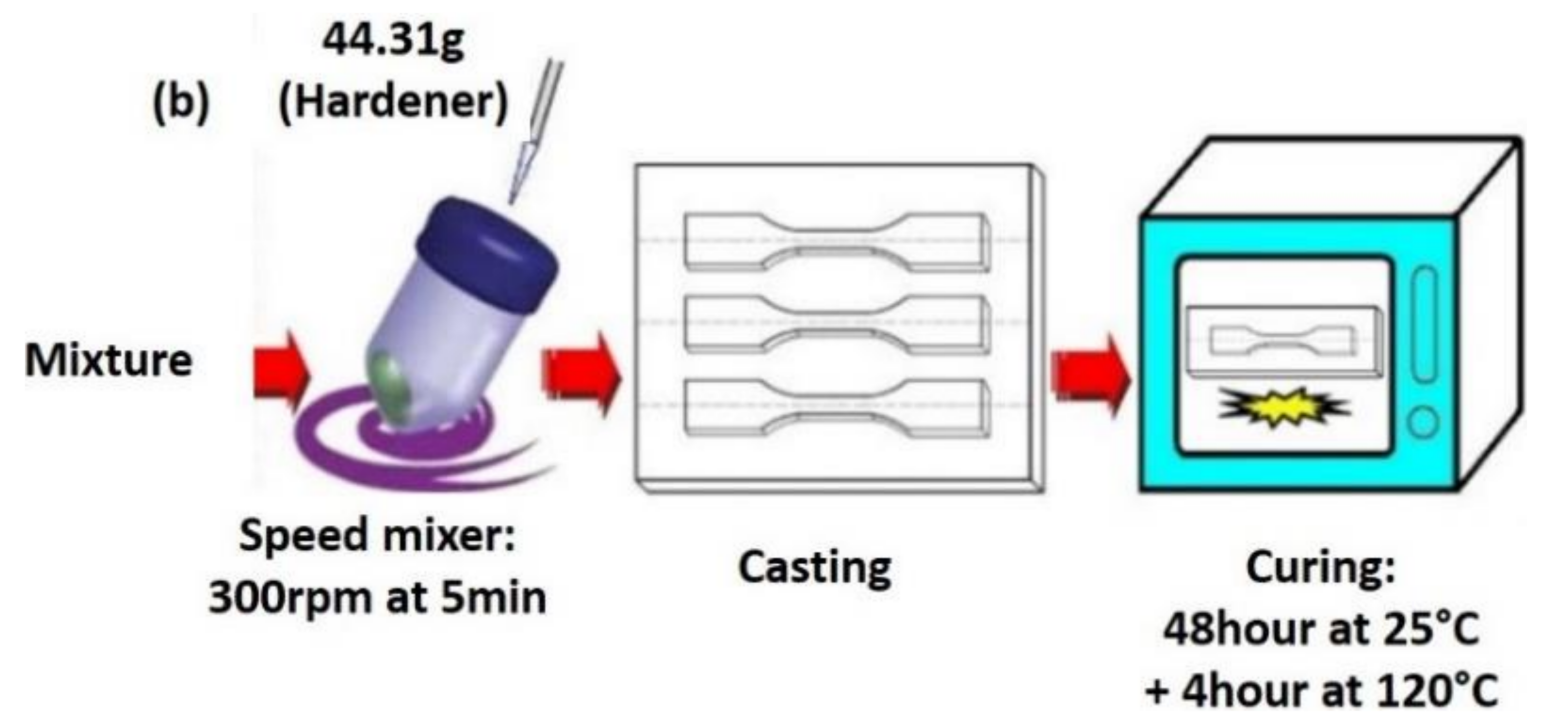
Schematic diagram of the fabrication process for the CNT–epoxy composites: (**a**) preparation of CNT suspension and (**b**) preparation of CNT–epoxy composite. Adapted from [96].

Sonication is widely used for dispersing CNTs in solutions, for functionalizing CNTs, and for formulating nanocomposites and nanomaterials [94,95]. In physical surface treatment, CNTs are dispersed in a solvent solution with the aid of dispersants and ultrasound before the addition of a polymer matrix like epoxy resin. During sonication, while the CNT bundles break down into individual CNTs, the dispersing molecules adsorb to the surface of the CNTs. Several factors, including the level of sonication treatment, the CNT geometry, type of dispersant had influenced an optimum dispersion of CNTs in solvent solutions and thus the efficiency of CNTs on the final composite structure. In other research, CNTs have been treated in a copolymer–ethanol solution to promote dispersion in the solvent and, consequently, in the epoxy resin. The process of mixing and dispersing CNTs in solvents and the manufacturing of CNT–epoxy composites is shown in Figure 4.

The energy level can be related to the threshold energy attributed to the sonication time and amplitude that yield the most optimized dispersion of CNTs and performance of composites. A lower concentration gives a higher threshold energy level [95]. However, a too strong sonication treatment can affect the rheological behavior of CNT-based composites, as an excessive sonication time can lead to the reduction of the nanoparticle aspect ratio, which counteracts the formation of a percolation network within the polymer matrix. According to Arrigo et al. (2018) [94], the reduction of the aspect ratio of CNTs can affect the thermo-oxidative resistance of nanocomposites, thus resulting in the deterioration of the long-term stability of the nanocomposites.

### 4.4. Resin Transfer Molding

**Figure 5:**
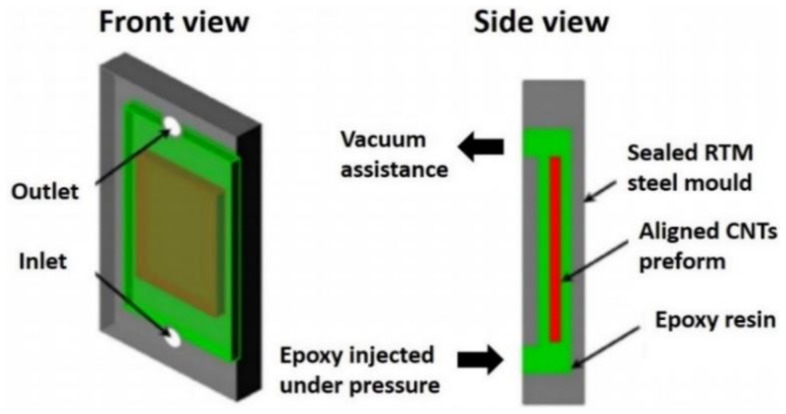
Schematic of the resin transfer molding process for fabricating CNT/epoxy composites. Adapted from [99].

Resin transfer molding (RTM) is another technique that can be applied to several types of CNTs with low-viscosity thermosetting polymers, and it can produce polymer composites with large sizes and complex shapes within a short cycle time and at a low cost. RTM allows for the manufacturing of versatile structures carried out in a closed system. Super-MWCNT arrays were synthesized with iron as the catalyst and acetylene as the precursor in a silicon wafer with a low-pressure chemical vapor deposition system [97]. From super-aligned arrays, CNTs were drawn and joined end-to-end by Van der Waals forces to create a continuous and aligned CNT sheet. To make a CNT preform, CNT sheets were stacked together at various orientations (Figure 5). The CNT preform was inserted into the resin transfer mold. The sealed resin transfer molding mold was injected with liquid epoxy resin and fully infiltrated the CNT preform in a vacuum oven. The oven temperature was then increased to cure the epoxy, forming a CNT/epoxy composite aligned with a solid-state. The resin transfer mold was then cooled down to room temperature, and the resin transfer mold was released from the CNT/epoxy composite sample. Composites may vary with the use of different CNT preforms and resin transfer molds [98].

### 4.5. Bucky Paper Resin Infiltration

Bucky paper offers a wide range of features that are capable of reinforcing existing multifunctional applications or invented new ones. Through this method, epoxy resin can be enhanced to become a high performance materials comparable to that glass fiber/epoxy composites with various properties including electrical insulation, semiconduction, thermal insulation, and thermal conduction [100]. An approach to making bucky paper-based phenolic resin composites was reported by Teotia et al. (2014) [101]. Firstly, CNTs were dispersed into a solvent and added to the dissolved phenolic resin. The suspension was then mixed using a high-speed homogenizer to disperse the CNTs in the phenolic resin mixture. The mixture was then filtered with a specially designed filtration system, and a film of CNT-impregnated polymer resin was obtained before being dried to get a CNT–phenolic resin prepreg. The prepreg was compression-molded using a hydraulic press. Since prepregs have become the main raw materials in the composites industry, this technique can be applied in composites science and technology for new materials with a combination of tailored mechanical, electrical, and thermal properties [100,101].

### 4.6. Aligned CNT Sheet Process

**Figure 6:**
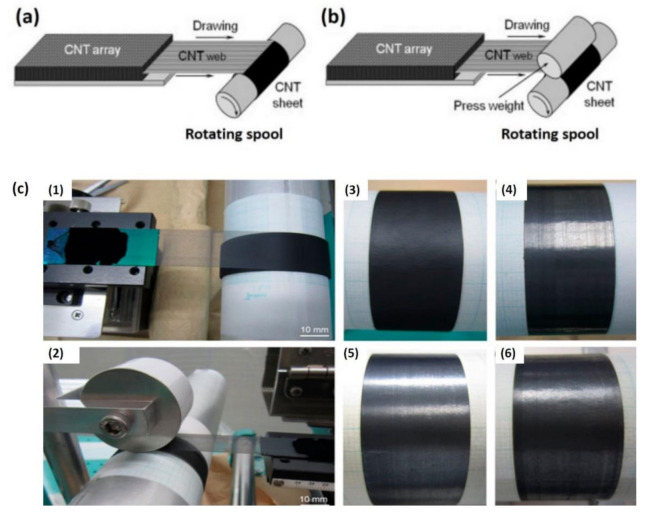
Schematic diagram showing aligned CNT sheet processing: (**a**) drawing and winding; (**b**) drawing, winding, and pressing (reproduced from [108]). (**c**) CNT sheet processing: (1) drawing and winding technique; (2) drawing, winding, and pressing process; (3) non-pressed CNT sheet; and (4–6) pressed CNT sheets under corresponding press load. Reproduced from [107].

High volume fraction CNT composites based on aligned CNT sheets have aroused great interest because they are envisioned for advanced composite materials for demanding applications [102,103,104,105,106]. However, achieving high volume fractions of dispersed CNTs in polymers is challenging due to the high viscosity that complicates further processing. Great efforts have recently been made to synthesize millimeter-scale aligned CNT arrays for the production of large-scale CNT structures. The aligned CNT sheets created by the solid-state drawing technique are lightweight and flexible [107]. The vertically aligned CNT arrays are self-directed, highly removable, and rotatable. Therefore, CNT webs are readily removed from arrays and wound to produce horizontally long-aligned CNT sheets on a rotating spool (Figure 6). For the manufacture of high-performance structural CNT composites, the press-drawing technique is effective in producing superior CNT sheets with the high alignment and dense packaging of CNTs [106]. However, the fabricated CNT sheet composites with aligned CNTs leads to limited improvement in electrical conductivity [108].

### 4.7. Shear Mixing

**Figure 7:**
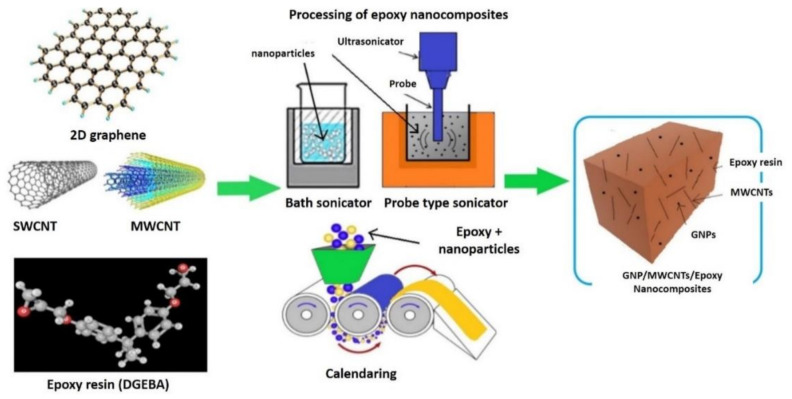
Schematic diagram for the shear mixing technique. Reproduced from [32].

Shear mixing has been most commonly used for thermoset polymers, e.g., epoxy resins. The calendaring process has been used in this technique. Three-roll milling is a general type of calendaring where the material is forced in between the rotating rollers, which inevitably mix it under a high shear force [109,110]. Compared to pure epoxy resin, graphene nano-platelet (GNP)/epoxy composites fabricated through the calendaring process have shown improved density, glass transition temperature, and thermal properties [32]. The three roller mill method and sonication combined with the high-speed scissor mixing technique to reinforce GNPs into epoxy matrixes has been conducted, as shown in Figure 7 [111].

### 4.8. In-Situ Polymerization

To solve the aforementioned dispersion issues between CNTs and polymers, it has been proposed to manufacture CNT composites via in-situ polymerization, especially for polymers that are insoluble, thermally unstable, and cannot be prepared by solution or melt processing [112]. This process is based on the dispersion of CNTs into a monomer matrix with or without the presence of a solvent, where a standard method of polymerization is then performed [113]. The advantage of this technique is that dispersion can be improved if the CNTs are provided with functional groups compatible with the monomer [113,114]. However, during synthesis, an insulating polymer layer is formed on the CNT surfaces, thus hindering the tunnelling mechanism within the composite [115]. As such, the strain sensitivity could also be negatively affected by the formation of a tunnelling barrier during in-situ polymerization [89].

In addition, in-situ polymerization is also used as a hybrid filler for nanocomposites for the physical functionalization of CNTs (through surface polymer wrapping). For the preparation of composites based on insoluble or thermally unstable matrix polymers, in situ polymerization remains the only possible option for materials that cannot be processed through the solution or melt processing routes. However, it is also used in other cases (where the above-mentioned limitations are not applicable) due to the superiority of in situ polymerization in terms of the ability to process high CNT-loading nanocomposites, to facilitate the good dispersion of CNTs within a polymer matrix, and to ensure excellent intimacy between CNTs and matrix polymers [90].

**Figure 8:**
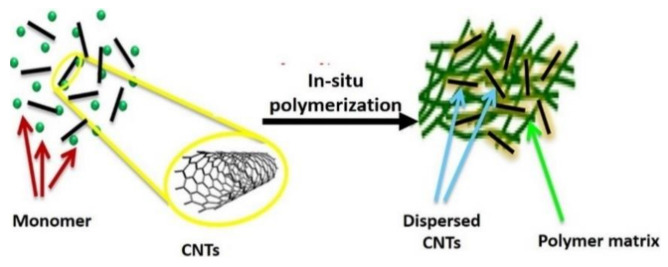
Schematic representation of in situ polymerization process. Reproduced from [90].

This process requires the dispersion of monomer CNTs (Figure 8), followed by in situ polymerization and leading to CNT/polymer nanocomposite formation. To enhance the distribution of the nanotubes in the monomer and, consequently, in the formed nanocomposites, the exploitation of functionalized CNTs or the use of monomer-grafted CNTs has been achieved. This has resulted in a stronger and more active nanotube–polymer interface that is central to the performance of nanocomposites for structural, electronic, electromagnetic, and electrochemical applications. Table 2 shows a summary of advantages and limitations of fabrication techniques for CNT-reinforced polymer composites.

## 5. Performance of Carbon Nanotube–Polymer Composites

Indeed, novel processing methods utilizing CNTs as potential composite fillers have improved the mechanical, thermal, and electrical properties of the resulting polymer composites [132,133]. Based on a report by Lourie and Wagner (1998) [134], one can observe that the mechanical properties of CNTs are dependent on the sp^2^ strength of the C-C bonds of the nanotubes, which makes CNTs good candidates as reinforcement fibers for matrixes. Interestingly, these types of bonding are even stronger than sp^3^ bonds found in diamonds. The carbon atom in a nanotube forms a planar honeycomb lattice, which is great at forming covalent bonds with other elements due to its electronegativity property. This electronegativity property is a crucial factor for the measurement of how strongly an atom holds onto the electrons orbiting around it. Because of their electronegativity properties, CNTs are expected to be strong enough to form stable covalent bonds to three neighboring atoms with various numbers of elements. It is theoretically known that the value of Young’s modulus for CNTs is around 1 TPa, which is approximately five times higher than that of steel [135,136], and the tensile strength varies from 11 to 63 GPa, which is around 50 times higher than steel [137,138]; both of these properties make them the strongest materials ever made by mankind. A comparison of mechanical properties between common structural materials like steel, aluminum, and different fibers is shown in Table 3 [139,140].

In addition to their mechanical properties, CNTs also have good chemical and environmental stability, as well as a high thermal conductivity of about 3500 W/m/K (which is comparable to diamond) [140]. This pronounced combination performance, coupled with the lightness of CNTs, gives them great potential to be applied in highly structural applications such as aerospace. Jiang et al. (2014) [104] studied the fabrication of CNTs with polyimide as the matrix in a unidirectional CNTs composite. When CNTs were incorporated into the polyimide, the modulus and strength of the composite increased by 12 times (nearly three times over pure polyimide), while, at the same time, the value of its electrical conductivity was multiplied by 10 times over pure polyimide. Furthermore, this CNT-reinforced polymer composite had an excellent thermal conductivity value with an increment of 600 times over pure polyimide, which is beyond what was achieved in a previous study. Table 4 shows a comparison of the mechanical, thermal, and electrical properties between pure polyimide and a CNT/polyimide composite [104].

**Figure 9:**
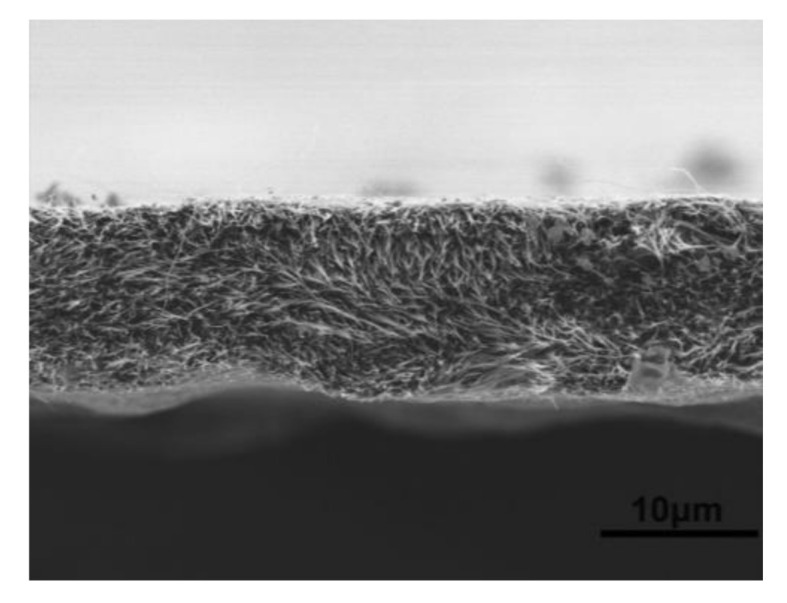
SEM micrographs of tensile fracture surface of CNT/polyimide composite. Reproduced from [104].

The dispersion of CNTs into the polyimide matrix was further investigated by means of microscopy. Figure 9 shows SEM micrographs of the tensile fracture surface of a CNT/polyimide composite. It can be observed that the composite was compact, probably due to the hot pressing and spray winding process. With the addition of CNTs, an interaction enhancement can be seen from the CNT/polyimide composite, which showed an improvement in uniform dispersion due to layer-by-layer winding.

Similarly, research conducted by Gardea and Lagoudas (2014) [141] on CNT/epoxy composites reported that the addition of CNTs into epoxy increased thermal conductivity up to 5.5%, and electrical conductivity improved by 10 orders of magnitude compared to pristine CNT/epoxy composites. Sankar et al. (2016) [142] observed an increment for tensile strength and Young’s modulus of 0.35 and 1.2 MPa, respectively, with the addition of 0.3 g of MWCNTs into an epoxy composite. Even with a small-quantity addition to polymeric composites, CNTs brought a positive contribution to the properties of mechanical strength and Young’s modulus compared with other high-performance synthetic fibers such as carbon fiber and Kevlar [143]. This was due to the better strength and adhesion achieved from long CNT length, the high level of CNT alignment, the good dispersion of CNTs in the polymer matrix, and the high CNT volume fraction [104].

In CNT/polymer composites, the properties of composites are greatly affected by the nature of bonding at the interface, the strength of the interface, and the mechanical load transfer from the surrounding matrix to nanotubes. The literature has also reported that the mechanism of interfacial load transfer from the matrix to nanotubes can be divided into two categories: the weak Van der Waals force between the polymer matrix and the CNT reinforcement [144]. In order to achieve a better physical and mechanical properties of CNT composites, the load stress must be effectively transferred from the matrix to the CNTs. Another factor that brings significant influence on the performance of CNT/polymer composites is the dispersion of CNTs in the polymer matrix through physical and chemical modifications [145].

Basically, the process of micro cracking takes place during curing or in-service at the fiber and matrix interface. Brittle resin systems are susceptible to micro cracking, especially at high processing temperatures and low service temperatures if there is a large difference of thermal expansion between the polymer matrix and the CNT reinforcements. Therefore, the existence of CNTs as a toughening reinforcement to a polymer resin matrix helps in preventing micro-cracking, though the performance at elevated temperatures is compromised at the same time [146]. However, limitations such as the agglomeration of CNTs often occurs due to the hydrophobic surface regions of the corresponding micelles surrounding the nanotubes. Therefore, a thorough understanding of the factors affecting the mechanical, thermal, and electrical properties of CNT–polymer composites has been an important focus of attention.

### 5.1. CNT-Reinforced Polymer Composites on Mechanical Performance

The CNT aspect ratio is a crucial factor in the longitudinal elastic modulus. Generally, CNT have a high aspect ratio, but their ultimate performance in a polymer composite is depends on the type of polymer matrix used. The effect of aspect ratio of CNT reinforcement on the Young’s modulus and yield strength was investigated in detail by Arash et al. (2014) [147]. According to their study regarding the effect of aspect ratio of CNTs on the mechanical properties of CNT/polymethyl methacrylate (PMMA), there was an increase on the Young’s modulus of PMMA polymer reinforced by CNTs, along with an increase of aspect ratio of CNTs; see Table 5. The diameter of the (5, 5) CNT reinforcements was 0.68 nm, and their length-to-diameter ratio (L/d) varied from 7.23 to ∞. Ultimately, a higher value of the aspect ratio of CNT increased the stress transfer between the CNTs and the polymer, which in turns led to the high strength and stiffness value of the CNT–polymer composites. It was found that the higher the aspect ratio of CNT, the higher the stress transfers from the polymer matrix to the dispersed CNT [148]. Thus, when there is a large enough aspect ratio of the reinforcement of CNT, there is an adequate load transfer through interfacial shear stress and the full strength of CNT can consequently be used.

Homogeneous dispersion is desirable because it enables uniform load distribution, therefore reducing the stress concentration and having great influence on the mechanical properties. The homogeneous dispersion of CNTs in a polymer matrix plays a crucial role in the preparation of polymer composites based on interfacial interactions between CNTs and the polymer matrix. Establishing a poor dispersion leads to the agglomeration when the load is applied beyond limit, resulting in lower values of mechanical properties [149,150]. This phenomenon is in agreement with the findings of a previous study carried out by Jia et al. (1999) [151] on the investigation of PMMA as a matrix, together with CNT reinforcement. It could be observed that the tensile strength, toughness, and hardness rose with an increase of CNTs ranging from 1 to 7 wt.%. However, the mechanical value decreased with the incorporation of CNTs up to 10 wt.%. This was due to the strong tendency of CNTs to form agglomerates due to their large surface-area-to-volume ratio value, which makes interfacial bonding a critical factor responsible for the overall properties of composites. Furthermore, when more CNTs are blended with a polymer, the CNTs remain as entangled agglomerates, which inhibits their homogenous dispersion in the polymer matrix. Similar findings were observed by Yang et al. (2007) [152], who emphasized that the incorporation of CNT loading from 0.2 to 1.0 wt.% into the epoxy matrix improved not only the impact strength properties but also the tensile strength. The impact strength of the CNT/epoxy composite improved with the addition of 0.2–0.6 wt.% loading of MWCNTs to the epoxy, but then the value of the impact strength decreased with the addition of 0.8–1.0 wt.% of CNTs.

The alignment of CNTs, even with a minimum amount of CNT loading (less than 1 wt.%), is the third factor for the contribution of mechanical properties, as well as another important factor in the identification of overall mechanical properties [153]. Random orientation and perfectly aligned CNTs demonstrate the different behaviors of mechanical properties. Aligned types of carbon nanotube polymer composites tend to behave anisotropically, which is not required for bulk composites. This type of alignment is technical relevant for the optimum mechanical properties due to its effect on stiffness and strength, but it is always beneficial. The alignment of CNTs was induced by shear forces during the melt extrusion and fiber drawing processes in the development of CNT/polyimide composites by Siochi et al. (2004) [154]. This alignment of CNTs further resulted in significantly greater tensile strength and modulus properties of a polyimide/SWCNT composite that is displayed in Table 6 [154]. Furthermore, another study conducted by Kearns and Shambaugh (2002) [153] confirmed that the increment of the mechanical properties of the composites was significantly increased from 709 to 1032 MPa, with the addition of small amount of CNTs between 0 to 1 wt.%, respectively.

The most crucial factor among all listed factors is interfacial stress load transfer. It is assumed that CNTs will disproportionally exploit the maximum load amount and impart a major load carrier, thus leading to delay in crack propagation. It is important to mention that there are three types of mechanisms dictated by the interfacial interaction between CNTs and polymers that are called mechanical coupling, physical interaction, and chemical interaction. Mechanical coupling involves the entanglement of polymers with CNT fillers, as well as the formation of micro mechanical lock, whereas physical interactions involve Van der Waals forces, and chemical interactions involve the use of functional groups for the efficient dispersion of CNTs in polymer matrix resin, polymer wrapping [155], and plasma polymerization [156]. Xie et al. (2007) [157] investigated the efficient interfacial stress load transfer between SWCNTs and polymer density covalent layers via the grafting method approach. The functionalized SWCNTs exhibited a remarkable impact on the mechanical properties of the polystyrene composites. The addition of only 0.06 wt.% of SWCNTs significantly impacted the tensile strength and elastic modulus of the composites, indicating that an efficient stress load transfer had occurred in this CNT/polymer composite [158].

Remarkable efforts to improve the mechanical properties of CNT–polymer composites have been made since the early 1990s. It is well-understood that the loading of CNTs is usually selected under 10 wt.% in order to avoid an increment in viscosity, which ends up resulting in a poor processability and weak properties of polymer composites. A summary of mechanical properties of CNT–polymer composites from 2015 to 2020 is given in Table 7.

### 5.2. CNT-Reinforced Polymer Composites on Thermal Performance

CNTs with a thermal conductivity an average of 10-fold lower than metals/ceramics have shown great success as good thermal insulators [90]. Furthermore, additional physical characteristics in terms of their light weight, cost-effectiveness, and corrosion resistance are very importance in the future trend of nanocomposite technology [176]. Interestingly, these characteristics can be envisioned as new possibilities for various applications of their thermal properties such as thermal interface materials, heat sinks, printed circuit boards, connectors, and other high-performance thermal management systems [90]. However, there is still a significant challenge faced among researchers of CNTs in terms of lowering CNT loading to provide a higher thermal conductivity [177]. 

The existence of shielded internal layer in MWCNTs could be promising for the conduction of phonon and the reduction of matrix coupling losses. These properties have shown that MWCNTs can provide the most significant improvement in thermal conductivity-reinforced polymer composites [90]. The extent to which MWCNTs correspond with the significant progress of thermal conductivity in polymer composites can be observed in Table 8. Not surprisingly, most polymers are combined with multi-walled carbon nanotubes to obtain a higher thermal conductivity depending on their application (Table 8). In addition, the affecting factors that influence the making of CNTs as very promising material in providing higher thermal conductivity also need to be studied to solve a significant challenge in CNTs, which is in lowering the loading value of CNTs to have a higher thermal conductivity [177]. Among the main factors influencing thermal conductivity is polymer morphology [178,179]. Markers for the prediction of higher thermal conductivity based on polymer morphology have been widely investigated, and it has been shown that more defects at the crystalline structures, the lower the intrinsic thermal conductivity of the crystal. This leads to phonon scattering, which is known to shorten the mean free path that finally reduced the thermal conductivity [176]. The morphology of CNT reinforced polymer composites significantly affect thermal properties. This can be observed through different mechanisms in the alignment of structure such as mechanical stretching [180], nano templating [178,181], and electrospinning [178,180,182]. Therefore, a clear understanding of the mechanisms involved in polymer morphology will contribute to the improvement of studies in designing a composite with a high thermal conductivity.

### 5.3. CNT-Reinforced Polymer Composites on Electrical Performance

One of the most exceptional properties of CNT-reinforced polymer composites that has brought them to another level of remarkable multifunctional materials is electrical performance. With CNTs, polymer composites have found significant value in electrical field applications such as solar cells, integrated circuits, sensors, aerospace, and shielding [192,193,194]. CNTs possess an electrical conductivity as high as 10^6^–10^7^ Sm^−1^ due to the fact that, even at very low filler loading, reinforced polymer composites with exceptional conductivity can be obtained [195]. Nanostructure size also plays an important role in inducing excellent conducting behavior through its ability to allow for homogenous embedding in polymer matrices. Here, various types of emerging organic polymers reinforce CNTs such as polyaniline (PANI)/ ≤ 8 wt.% f-MWCNT with an electrical conductivity of about 28.6 Sm^−1^ [196], polyamide/7 wt.% MWCNT with an electrical conductivity of ~10^1^ Sm^−1^ [194], PVDF/0.5 wt.% MWCNT with an electrical conductivity of about 0.8 Sm^−1^ [197], PC/ ≤ 3 wt.% SWCNT with an electrical conductivity of about 10^1^ Sm^−1^ [198], and polypropylene (PP)/ ≤ 3.5 wt.% MWCNT with an electrical conductivity of about 2.0 × 10^1^ Sm^−1^ [193] were reviewed to study this property. On this subject, studies have focused on the mechanisms and factors that facilitate and affect electrical conductivity in the CNT-reinforced polymer composites.

#### 5.3.1. Mechanism of Electrical Conductivity

**Figure 10:**
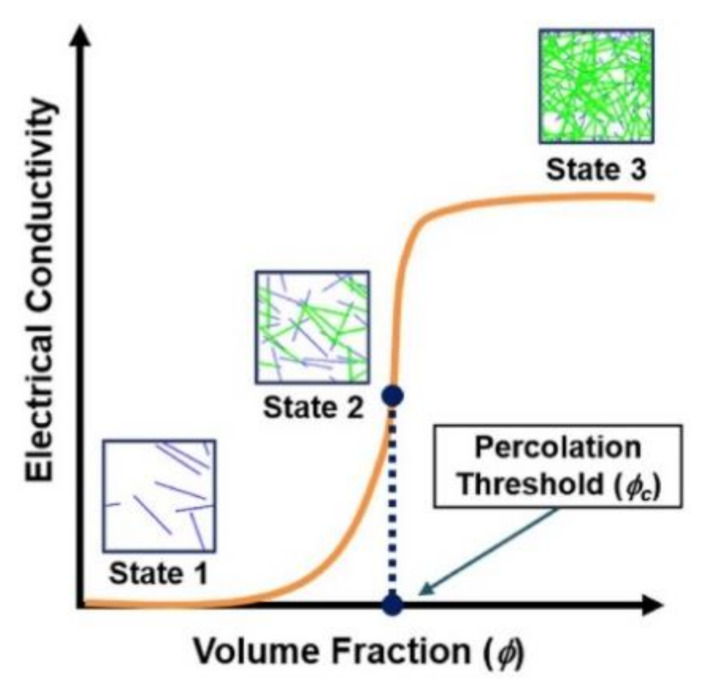
Classifications of three different states concerning the percolation theory-based electrical conductance transition for CNT-filled polymer nanocomposites. Reproduced from [204].

Man reports [199,200,201,202,203] have indicated that the incorporation of CNTs with polymers leads to a lower percolation threshold due to the high aspect ratio and intrinsic conductivity of CNTs. The percolation threshold in a polymer matrix composite is the minimum content of filler that allows for no changes in the electrical conductivity of the composite. As such, when the concentration or weight % of the filler exceeds the percolation threshold (upon stipulated states), conductivity increases (Figure 10) [204]. The introduction of CNTs into a polymer matrix lowers the percolation threshold because the nanotubes are covalently incorporated into the polymer cross-linked structure. This network structure of fillers create conductive paths where the CNTs are in close contact to each other, thus facilitating the conduction of electron through ‘hopping’ or ‘tunnelling’ mechanisms [100]. Therefore, with increased volume fractions of CNTs, the network paths intensify and hence abruptly increase the conductivity of reinforced polymer matrices. Experimental approaches based on this interesting mechanism of dispersion are further discussed in the next sub-section of factors that affect the electrical conductivity of CNT-reinforced polymer composites (filler loading amount).

There have been supportive studies on percolation theory [205,206,207] that discussed the importance of dispersion quality in assisting the increment of electrical conductivity which, is also supported by theoretical models. It has been established that there are three main mechanisms of conductivity in percolating CNT networks: (1) the electrical conductivity of the CNTs themselves, (2) the direct contact conductance at the microscale, and (3) the nanoscale phenomenon of electron hopping or tunnelling. These three mechanisms of conductivity have become the guidelines for proposed models for the quantitative prediction on electrical conductivity of CNT-reinforced polymer composites [202]. The Monte Carlo method has been widely used to investigate the electrical percolation behavior of CNT-reinforced polymer composites. The method has allowed for the evolution of numerical predictions with unlimited forms of filler structures, whether powders or films with wide ranges of dimensions. Ni et al. (2018) [208] applied this method in their study to come up with numerical predictions of the percolation threshold of an insulating thin film that was reinforced with 1D and 2D conductive fillers. Mechanism parameters such as size effect, concentration, and contact patterns of nanofillers were carefully examined for the prediction. Their prediction showed that different dimensions of nanofillers can coexist and give a synergistic effect to obtain an effective conductance.

#### 5.3.2. Factors Affecting Electrical Conductivity of CNT-Reinforced Polymer Composites

There are many factors that affect the electrical conductivity of CNT-reinforced polymer composites. Significant factors that have been identified from theoretical models and experiment works are (1) the dispersion of CNTs, (2) the filler loading amount, (3) the structure of CNTs, and (4) the type of polymer. 

##### Dispersion of CNTs

In an experiment, Gao et al. (2018) [209] prepared incorporated CNTs with polyurethane (PU)/polyethersulfone (PES) nanofibers using ultrasonication, which resulted in highly dispersed CNTs on the nanofiber surface upon ultrasonication time. The dispersion constructed electrically-conductive networks with an ultralow percolation threshold of 0.056 vol.%, which led to a significantly high electrical conductivity at 2.8 Sm^−1^ at a relatively low CNT concentration of 0.85 vol.%. Moreover, with the aim to improve the epoxy resin’s electrical performance, Trakakis et al. (2020) [100] produced CNT/epoxy nanocomposites using the bucky papers approach. This approach allowed for better CNTs dispersion into the matrix with longer CNTs, which improved the total conductivity of the nanocomposites. The report showed a significant increase of the electrical conductivity (from 0.17 to 0.57 Sm^−1^) of the insulated epoxy resin to a level that was comparable to semiconductors.

##### Filler Loading Amount

Recently, Chen and Han (2020) [200] focused on identifying the causes that may lead to an improvement of the electrical conductivity in reinforced, CNT-derived epoxy polymers. The findings led them to the agreement of the electrical percolation threshold theory. They found that the electrical percolation threshold existed in all their derived composite materials, which exhibited increments of electrical conductance upon the increased concentration of CNTs. They found that the conductivities increased by up to 10^1^ Sm^−1^ when the CNT content increased from 1 to 5 wt.%.

Segregated conductive polymer composites (CPCs) possess a very low electrical percolation threshold (1.62 wt.%) because the segregation provide pathways for electron conduction. However, CPCs suffer from a poor mechanical strength and brittle nature. Concerned about this problem, W. Zhai et al. (2018) [210] developed an easy technique to fabricate CPCs. They introduced plunger-type injection molding (PTIM) to sustain the segregated structure while simultaneously improving the mechanical strength. Their effort showed a positive hypothesis, because the resistivity of all samples exponentially decreased with the increasing CNT content. However, the decrease in resistivity explained the increase of conductivity. Furthermore, an impressive threshold percolation of about 0.13 vol.%, which was 30 times lower than the specimens gained from the conventional injection, was obtained. 

##### Structure of CNTs

Researchers have been more attracted to MWCNTs than SWCNTs. This is because the densities of MWCNTs are higher than SWCNTs, which makes MWCNTs possess lower surface areas and higher aspect ratios. These enable good dispersibility, hence increasing the electrical conductance of polymer matrixes. With the application of an externally applied field, Gupta et al. (2016) [197] showed that a CNT-incorporated PVDF matrix significantly increased conductivity by a huge order of 10—10^5^ Sm^−1^ for CNT/PVDF compared to only 10^−5^ Sm^−1^ for PVDF. Additionally, the method of preparation through stirring and ultrasonication helped to provide a better dispersibility of CNTs, which is another factor that contributes to a high conductivity. Later, T. Yamamoto and K. Kawaguchi [211] produced a copolymer (styrene monomer, benzyl methacrylate and methyl methacrylate) via in situ polymerization. To obtain a high dispersibility, they dispersed MWCNTs with very fine membranes (100 nm pores), which ensured the formation of conductive paths and thus enabled a significant conductivity result of 1.2 Sm^−1^ with only 0.5 wt.% of CNT content.

Attractive novel MWCNT/phosphate glass (Pglass)-reinforced PP hybrids were proposed by L. Zhang et al. (2018) [193] to come out with highly controllable dispersibility and fine morphology nanofillers. It is well-known that hybridization may lead to new property outcomes while maintaining existing ones, which is a strategy to diversify matrix applications. Typically, Pglass is composed of inorganic semiconductor oxides such as SnO, P_2_O_5_, and SnF_2_. Uniquely, it possesses polymer-chain-like molecular structure with an ultra-low glass transition temperature (40–200 °C), hence allowing for homogeneous blends of Pglass and polymer for controllable dispersion with a tuned morphology of Pglass during processing. With the increased content of CNT/Pglass in the matrix, the electrical conductance greatly improved to as high as 21.9 Sm^−1^.

Despite having less attention, SWCNTs play important roles in more specific application such as the aircraft industry. Their lesser density property allows for the production of a light weight matrix, as proven by Q. Xia et al. (2020) [212]. They produced a highly conductive silver modified CNT film via electrophoretic deposition (EPD) for protecting carbon fiber-reinforced polymer structures and components of aircraft. In comparison to conventional metallic materials, the utilization of CNTs reinforced polymer composite decreases aircraft fuel consumption and increases fatigue resistance. This significant improvement is evident in Boeing 787 body structures and components built using 50% of CFRP composite by weight.

##### Type of Polymers

As summarized in Table 8, polymers exist in two main groups: thermoplastic and thermoset. Both have been used as polymer-based matrices for engineering polymer nanostructures. Consequently, thermoplastics have been found to have low melting points (high solubility), while thermosets can withstand higher temperatures without a loss of their structural integrity (superior strength). Their electrical performance is discussed below.

(a) Thermoplastic polymers: PP is known to be one of the highly favored thermoplastic polymers due to its high solubility in aqueous media and low cost. Being a thermoplastic commodity, PP has the advantage of recyclability with remarkable physical, thermal, and mechanical properties. PANI also has similar advantages and has been a subject of reinforced polymer research for many years [213,214,215]. Sobha et al. (2017) [196] successfully produced highly disperse functional MWCNTs (f-MWCNT) in thermoplastic polyurethane (TPU) composites based on PANI via an in-situ polymerization assisted by ultra-sonication. The well-dispersed and reduced aggregation of f-MWCNTs contribute to a very low percolation threshold at only 0.58 wt.%, hence exhibiting a significant conductivity of 28.6 Sm^−1^. The results showed promising applications, mostly as coating and electromagnetic interference shielding.
(b) Thermoset polymers: As a conducting polymer, polypyrrole (PPy) has been widely used due to its superiority in optical and electrical properties. It possesses a high efficiency response towards visible light, a high carrier mobility, and an excellent thermal and chemical stability. Recently, by looking into these advantages, Saheeda and Jayaleksmi (2020) [216] successfully synthesized a nanocomposite of PPy and MWCNTs via liquid/liquid interfacial polymerization. In this study, they found that a strong interfacial interaction between the polymer and MWCNTs occurred. This led to the establishment of a high electrical conductivity of 8.05 × 10^2^ Sm^−1^. Furthermore, the nanocomposite also possessed excellent nonlinear optical properties that suggested promise for its application in solar cells. Table 9 presents the properties of the electrical conductance of various CNTs and polymers, as well as their potential applications.

### 5.4. Environmental Concern and Health and Safety Issues of CNTs

The toxicity and health and safety issues presented by some forms of the material from CNTs depend on several factors like aspect ratio, length, surface area, degree of aggregation, purity, and concentration [221].

#### 5.4.1. Aspect Ratio

The difference between CNTs and other types of commercial reinforcing fillers including carbon black, clays, and carbon fibers is that a better compatibility of CNTs with polymers could be obtained with smaller aspect ratio CNTs, representing the formation of more uniform CNT/polymer composites. The high aspect ratio of CNTs means dynamic light scattering techniques are unsuitable for generating accurate particle sizes in dispersion [222]. Thus, it has been proposed that for the determination of more relevant particle size data and particle size distributions of CNTs, SEM and TEM imaging are better options. Therefore, in order to ensure health and safety while exposing CNTs to the environment, the occupational exposure limit values (OELs) standard had been conducted as legislation applicable to nanomaterial handling. Information regarding OELs for nanomaterials is presented in Table 10 [223].

#### 5.4.2. Length

Several experiments have been conducted to study the relationship between different lengths of MWCNTs and pulmonary fibrosis [224]. Based on the obtained results, long MWCNTs have much heavier adverse pulmonary effects compared to short MWCNTs. The size and the composition of a nanomaterial play a major role in the cellular response, which is related to the physiological function of the cell [225]. Observations showed that inflammation, fibrosis, and angiogenesis can be triggered by MWCNTs due to their length, iron contents, or crystal structure [226].

#### 5.4.3. Surface Area

Another critical aspect that has been pointed out as a factor of toxicity is the surface area of CNTs. In a study by Kim et al. (2010) [227], the toxicity of a nanomaterial was found to be highly affected by its physical properties, like the size distribution and surface area reactivity of particles. In bronchoalveolar lavage fluid (BALF) cell analysis, pristine multiwalled carbon nanotubes (pMWCNTs) induced more severe acute inflammatory cell recruitment than acid-treated multiwalled carbon nanotubes (tMWCNTs). This was due to the fact that the reduction in size to the nano scale increases the surface area ratio of the materials and therefore increased the potential to cause damage; but this phenomenon was not possible while they were in larger forms [228].

#### 5.4.4. Concentration

In order to further understand the relationship between CNT behavior and toxicity, concentration is another critical aspect that needs to be studied. In a study on mice that were exposed to the pharyngeal aspiration of purified pristine SWCNTs, the dose-dependence and time-course of pulmonary responses were examined [229]. Based on the obtained results, the SWCNTs produced acute inflammation, progressive fibrosis, the formation of granulomas, and an increase in protein levels, all of which were validated and detected.

#### 5.4.5. Cost

It is well-known everywhere that costs comprise another issue that is related to health and safety. A cost-effective solution can be a factor worth considering for handling the health and safety issues related to CNTs. The evaluation of cost efficiency must be done with a holistic approach. In order to implement the final calculation of the cost of safety of a nanomaterial, a list of the main parameters was designed and implemented [230]. A list of main parameters included costs related to raw materials, equipment, insurance, security, decommissioning, and software. This practice was applied in the development phase, giving guidance to the technology selection and the subsequent improvement of safety and sustainability [231].

## 6. Applications and Potential Use of Carbon Nanotube-Reinforced Polymer Composites

Polymeric composites are one of the most well-known materials that have lightweight properties and high durability for various functions [232,233,234,235,236,237,238,239]. Polymer composites exploit a wide range of applications due to their all-around excellent performance in mechanical, thermal, and electrical properties [240,241,242,243,244,245]. The inclusion of nanofillers inside polymer resins could provide promising properties for materials in almost every sector [246,247,248]. Due to high cost of carbon fibers, CNTs can be added in small quantities in polymeric composites but exhibit while exhibiting strong mechanical properties [249]. For instance, the application of CNTs as reinforcements in polymeric composites could establish significantly high mechanical strength and elastic modulus values in comparison other high performance fibers such as Kevlar and carbon [143,250,251]. To be specific, their tensile strength and elastic modulus have been recorded at 150 GPa and 1 TPa, respectively, which marks them as tremendously stronger and stiffer, as well as three-to-five times lighter, than steel. These properties can be characterized by using a proper testing facilities [252,253] to ensure the qualities are on par with current conventional materials. Since these materials have shown significant enhancement in term of their material properties, Table 11 summarizes recent research on CNT–polymer composites conducted in various sectors.

**Figure 11:**
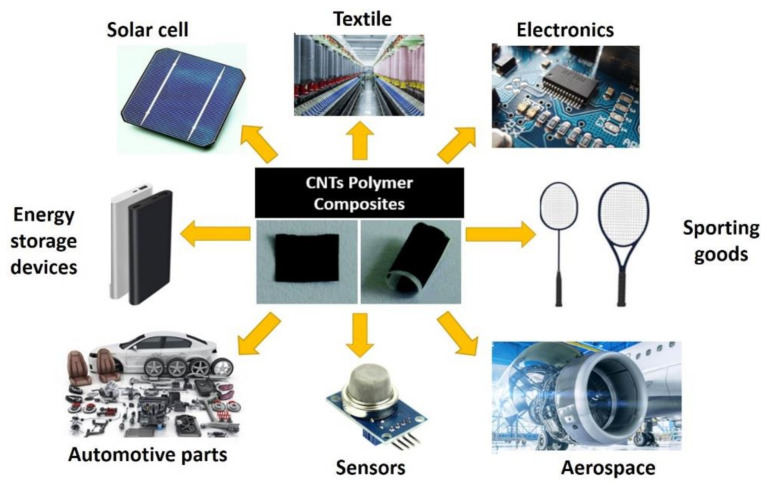
Application of CNT–polymer composites.

CNTs are emerging advanced materials with outstanding mechanical, electrical, and thermal properties and highly interfacial contact areas. in comparison to other polymers, cnt–polymer composites have received more attention among material scientists due to the good compatibility between cnt and polymers. Figure 11 shows the potential and current applications of CNT–polymer composites including electronics, automobiles, textiles, aerospace, sport equipment, sensors, energy storage devices, and filters [260,261,262].

### 6.1. Electronic Application

The advanced applications of CNT–polymer composites are rising quickly in the electronic field, especially in the development in electronic devices. The growing demand for advanced materials with customize electrical properties makes CNTs the most attractive nanomaterials for electrical and electronic devices. The enhancement of field emission properties can result in the improvement of the efficiency of electronic devices. Connolly et al. (2009) [263] evaluated the field emission properties of CNT–polymer composites produced by solution processing. They found that an excellent electron emission could be obtained at a 0.7% volume fraction of nanotubes in a composite. The study also established that a good combination of the type of polymer and the concentration of CNT could improve the charge transfer through the composite. Likewise, research conducted by Jin et al. (2006) [264] showed that the triode-type field-emitting arrays could manufactured from CNT-reinforced PPy composites. They stated that CNT–PPy composite devices showed a better Fowler–Nordheim characteristic, which could potentially help their use in the semiconductor industry. More recent research conducted by Gupta et al. (2014) [265] found that the field emission properties of CNT/PPy nanocomposites were enhanced due to improvements of the electronic properties of PPy on the CNT layer, even when no dopant was added during the synthesis of PPy.

Additionally, CNT–polymer composites are useful when preparing solar cells. According to Sibinski et al. (2011) [266], elastic CNT–polymer composites have a high potential to produce new photovoltaics through a screen-printing technique. They discovered that the CNT–polymer composite had a high optical transmittance with less costly manufacturing process. The nanocomposite had a better elastic behavior and significantly strong optical and electrical parameters, which gives them potential use as coatings in solar cells.

### 6.2. Aerospace Application

The aerospace industry requires very high strength and durable materials to be embedded as components in astronautic equipment. Since CNTs has various characteristics, the materials have been widely studied to evaluate their potential to act as constituents of composite materials. The CNT–polymer composites are highly suitable for the aerospace and aeronautical fields. For the aerospace field, the CNT–polymer composites have been actively studied by researchers in order to enhance the electrical performance of composites with epoxy resin. Thus, CNT–polymer composites are essential in the aerospace field due to their structural properties that could be applied to such areas as in anti-radar protectors, antistatic materials, and spacecraft [267]. CNT-reinforced epoxy polymer composites have been commonly utilized in air/spacecraft developments since 2006. CNTs are emerging advanced materials that allow a structure to be lightweight, have elevated temperature resistance, and have high strength-to-weight ratio. Study by Kim et al. (2011) [268], tensile tests were conducted at 25% from their yield strength, and 4 wt.% CNT-reinforced polymer composites was discovered to break. In general, the 1 wt.% CNT composites doubled the Young’s modulus and yield strength compared a pure epoxy laminate, as shown in Table 12.

In accordance with the work of Belluci et al. (2007) [269], CNT–polymer composites can exhibit significant changes in their resistivity value, which is important for high-fidelity circuits in aerospace application. Additionally, the technology of electromagnetic interference (EMI) shielding was developed with CNT–PP composites by Al-Saleh and Sundararaj (2009) [270]. They indicated that the shielding from CNT–polymer composites provides absorption (major shielding mechanism) and reflection (secondary shielding mechanism). Moreover, the EMI shielding effectiveness of CNT–PP composites was elevated with increases in CNT content and shielding plate thickness, which showed the efficiency of the CNT nanocomposites. Next, CNT reinforced polymer composites could act as heat absorbing media that are useful in aerospace industries, e.g., as electromagnetic wave absorption materials [271]. 

### 6.3. Automobile Application

In the automotive sector, nanocomposite materials—especially CNT–polymer composites—could be beneficial in many ways, including the improvement of existing technologies. CNT reinforced polymer composites could be applied to automobile parts including exhaust systems, catalytic converters, suspension and breaking systems, electronic equipment, engines, power strain materials, and body parts [272,273]. Previously, traditional fillers such as mica, calcium carbonate, and talc were widely applied in automotive parts in order to offer higher melt viscosity, optical clarity, and better stiffness properties. For instance, glass fiber was introduced due to its high in stiffness, but it is difficult to fabricate and thus incurs high production costs. Additionally, traditional fillers and glass fibers have to be implemented with high loading to improve dimensional stability, increase the mechanical modulus, and increase surface quality. Thus, the introduction of CNT–polymer composites in this industry could aid the aforementioned issues of traditional fillers.

CNT fillers are effective at lower concentrations (0.2 wt.%) in polymeric composites because they can significantly enhanced dimensional and thermal stability, as well as reduce weight [259,274]. CNT–polymer composites also play significant roles in automobile engineering. CNT–polymer composites have been found to possess a high strength-to-weight ratio because a lightweight vehicle could allow for a vehicle to have a lower fuel consumption. This would result in the reduction of carbon dioxide emissions by the vehicle, which could help to reduce global warming. Yang et al. (2012) [275] discovered that a 25% reduction in vehicle weight would reduce crude barrel consumption by up to 250 million barrels per year. Many car manufacturing companies have employed nanocomposites in trunk lids, car seats, dashboard coverings, and roofs [276].

Furthermore, the addition of MWCNTs in epoxy composites would increase the adhesion strength of the matrix, which would subsequently contribute to lower water intake, hydrophobicity, and corrosion resistance [277]. Another study conducted by Lee et al. (2010) [278] found that the inclusion of CNTs and montmorillonite in epoxy resin would permit good anti-oxidation and flame retardant properties. These findings showed that CNT–reinforced rubber composites have a high potential to produce high-performance vehicle tires. According to Jia and Wei (2017) [279], the application of CNT rubber composites in tires would induce a high thermal conductivity and a low hysteresis. This would cause the tread base and shoulder parts to reduce the heat accumulation, which would subsequently prolong tire durability.

### 6.4. Sensors

CNT reinforced polymer composites have the significant ability to detect chemicals in the air for various purposes. Because they allow for the good ability to sense gas molecules, they would benefit space exploration; environmental monitoring; and medical, industrial and agricultural applications. For instance, the detection of carbon monoxide, nitrogen oxide, and ammonium is required to monitor environmental pollution in the industrial and medical environments. According to Kong et al. (2000) [280], individual SWCNT composites have been established in chemical sensor applications. It was discovered that the exposure to chemical molecules such as nitrogen oxide and ammonium of semi-conducting CNTs would provide changes in electrical resistance. Currently, electrical sensors implement carbon black polymer composites. This shows that the CNT–polymer composites could provide better and faster responses than current materials. Another study carried out by Sattari et al. (2014) [281] discovered that methane gas was efficiently sensed by CNT/polyaniline composites at room temperature. Likewise, Rajabi et al. (2013) [282] applied CNT/polyvinyl chloride (PVC) mixed matrix membranes for gas separation applications. Khan et al. (2014) [283] also established that similar nanocomposites that can function as indicator electrodes for titration of the potentiometric materials.

### 6.5. Sporting Goods

The promising values of CNT–polymer composites in this modern era have led many material scientists and engineers to conduct various studies in many study areas. One of the most stimulating characteristics of such composites are their light weight, high strength property, and strong stiffness property, which render them superlative fillers [284]. Due to the fact that CNT–polymer composites have high stiffness and strength values, the nanocomposites have been turned into structural products and applications such as civil engineering structures and sporting goods [285]. For superior composite sporting goods such as badminton rackets and golf sets, epoxy has been used to reinforce the CNT fillers. As such resins of this class have excellent specific strength, stiffness, chemical resistance, and dimensional stability [232,235,237]. However, there are still many challenges for CNT-reinforced thermosetting polymer composites. These issues include the development of material features of nanocomposites when transferring the mechanical, thermal, and electrical properties of CNTs to epoxy composites [286].

### 6.6. Wind Turbine Blades

The renewable energy sector is currently growing rapidly to replace conventional energy such as coal and petroleum. One of fastest growing energy production sectors is wind energy. According to the US Department of Energy, the country aims to generate “green” energy as at least 20% of its total energy needs with wind-generated electricity by 2030. Thus, this industry intends to produce a high efficiency and optimum production of energy by generating large blades that are lighter in weight. This is due to the fact that the production of wind energy increases with the square-area of rotor radius [287,288]. The current goal in the field is to produce larger wind blades with good mechanical properties, light weight, and long fatigue life. However, this goal is huge challenge for many researchers as most lightweight, high strength, and high stiffness materials would have high costs in raw material and production.

In order to overcome this issue, a CNT-reinforced polymer matrix is potential material to be implemented in the production of wind blades. Based on previous studies, the application of CNT fillers as strengthening agents has highlighted the influence of CNTs on the stiffness and strength of composites. More recent research found that the inclusion of CNT fillers in composites could enhance fatigue resistance and subsequently prolong fatigue life [289]. Thus, many researchers are recently working on CNT-reinforced thermoset polymer composites in order to enhance tensile and fatigue properties for wind blade applications. In general, epoxy polymers are not suitable for the large scale production of wind blades because they have a shorter fatigue life and poorer fracture toughness. This, in turn, limits the operating life and reliability of wind blades in long term use. Thus, studies have to focus on the long-term prospective of CNT–polymer composites under cyclic loading to be utilized for structural applications that require an increased fatigue life.

According to Böger et al. (2010) [290], the inclusion of 0.2 wt.% in epoxy polymer could enhance the fatigue resistance of composites. This could be done by dispersing the CNTs throughout epoxy resin with help from copolymers via sonication. Moreover, those tensile and dynamic mechanical properties were evaluated for pure epoxy and CNT–epoxy composites with five different load levels (25, 30, 40, 45, and 50 MPa). At the end of the experiment, it was established that CNT/epoxy composites exhibited a long fatigue life and significant improvements of fatigue properties in high cycles. Moreover, the improvement in fatigue life occurred due to the pull-out of the CNTs and crack bridging at the crack interface, thus showing that CNT-reinforced polymer composites would be the most promising candidates with high fatigue lives to be used in major structural and dynamic applications.

### 6.7. Environmental Remediation

Globally, the increase in the pollution rate due to urbanization and industrialization has caused tremendous negative effects on environmental ecosystems [291,292]. Flora and fauna can be adversely affected by various types of contaminants such as chemical, physical, radiological, and biological contaminants [293]. Water contamination has become a worldwide problem over past few decades because of the disposal of contaminated waste in water systems. Preventative measures have to be implemented in order to reduce catastrophic effects on the environment.

In this case, the application of CNT–composites is one way to remedy excessive environmental pollution. CNTs have special adsorption capacities for different types of environmental pollutants by a large accessible external surface area, a high aspect ratio of fibrous shapes, and strong electrostatic interactions with charge pollutants in water [294]. In detail, CNTs can absorb pollutant particles on their external surfaces, open-ended portions, groves at the line boundary of carbon nanotube bundles, and the interstitial pores among the tube bundles [295]. It can be seen that CNTs have a good membrane separation ability that is especially useful for water treatment processes. CNT surface structures also have cytotoxic effects that inhibit the growth of microbes. Nanofillers have also been shown to contribute self-cleaning properties to CNT filters [296]. CNTs also considered to be good catalyzers for immobilized enzymes. In this case, immobilized enzymes on CNTs have shown more stability, broad pH ranges, more storage stability, better capacitive deionization, and more reusability. Yan et al. (2011) [297] successfully removed aniline aromatic compounds from water molecules by implementing immobilized enzymes of *Delftia sp. XYJ6* on CNTs. Zhai et al. (2013) [298] showed that CNT–horseradish peroxidase enzyme could remove phenolic compounds from polluted water. Additionally, CNT-reinforced polymer membranes have a good ability for diffusivity, which makes them highly significant for water purification and adsorption systems for heavy metals ions, small molecules, organic chemicals, and radionuclides [299].

## 7. The Economic Analysis of CNT Production

The production of CNTs is anticipated to increase due to countless utilizations and applications in advanced nanotechnology. In 2018, the CNT market was estimated to grow from USD 4.55 billion to 9.84 billion by 2023 at a compound annual growth rate (CAGR) of 16.70%. The growth of the CNT market is affected by the cost structure of CNTs, processing difficulties, and the availability of substitutes or specific functional groups such as silicon carbide nanotubes (SiCNTs). The methods that are used to produce carbon nanotubes, such as arc-discharge and laser ablation methods, are complex, expensive, hazardous for nature, and uneconomical for the production of CNTs on a large scale. The Asia Pacific Countries (APAC) carbon nanotubes market is expected to grow at the highest estimated CAGR during the forecast period. This is attributed to the robust demand for CNTs in India, China, South Korea, Vietnam, Taiwan, and Singapore; China is currently leading the demand for CNTs due to its increased industrial production, and it is largest consumer of CNTs at the global stage. The demand for CNTs has been rapidly increasing in applications such as field emission displays, integrated circuits, hydrogen storage, lithium (Li) batteries, solar photovoltaic (PV) cells, fuel cells, chemical sensors, and drug delivery. Increasing commercialization; the ramping up of installed capacities; and technological advancements to lowering the price, improve quality, and develop more advanced products are the trends in the CNT market. This includes in the demand for CNTs in the aerospace and automotive industries in order to make aerospace components stronger, tougher, and longer-lasting. The industry is attempting reduce weight in an ongoing bid to reduce fuel consumption and, by extension, operating costs. CNTs may be utilized in the aerospace and automotive industries for improved (or tailored) characteristics that improve their functional performance, especially their mechanical or electrical properties or those deliver multi-functional properties like light weight and conductivity. Furthermore, CNTs with distinct 1D-tubular structures, excellent electrical and thermal conductivities, and significantly large surface-areas are considered to be best nanomaterials to enhance Li battery performance. Li batteries are expected to have the highest market share during the forecast period from 2018 to 2023. The demand for Li-ion batteries is rapidly increasing in vehicles that require light weight and high-energy density solutions for batteries. These batteries provide the highest energy density per weight and are widely used in cellular phones, notebook computers, and hybrid automobiles [300].

## 8. Conclusions and Future Outlooks

The addition of CNTs to polymer composite structures with natural fiber has opened a new era of polymer composites for various structural applications. As polymer matrix reinforcements, different types of CNTs with specific and unique functional groups interact with hydroxyl groups in natural fiber cellulose chains, thus modifying the natural fiber surface. This might provide new promising interfacial bonding features. Definitely, among the applications related to CNT-based materials, the incorporation into natural fiber/fabric polymer composites has very recently attracted special attention for wide-ranging applications. Outstanding performance has justified the rapid increase in publications and has motivated attempts to develop engineering products. Indeed, from the aforesaid exponential growth, it is predicted that around 3000 articles on graphene-incorporated natural fiber polymer composites might be made by 2025 [301]. The incorporation of CNTs into polymer composites has been shown to significantly improve the strength, stiffness, toughness, electrical conductivity, and thermal stability of composites. Based on the articles discussed in this review, one should expect a promising future perspective for research works on the incorporation and functionalization of natural fibers with CNTs for a new generation of high-performance composites. Alternatively, there are relevant technological challenges to overcome, such as CTNs’ reproducibility in large-scale applications, as well as their homogeneous dispersion in composites and their interfacial interactions. The safety, reliability, and durability of these composites are still subject to exploration. There is no hesitation that CNT hybrids with natural fiber polymer composites should promote pioneering research works and contribute to industrial developments, but more fundamental studies are needed to provide a better understanding of the interaction between components in this surging, novel class of polymer composite materials. From that, a prediction on the overall chemical interaction and response of bulk composites due to the existence of multiple interfaces (CNT/polymer interface, natural fiber/polymer interface, and CNT-modified natural fiber/polymer interface) requires the critical understanding and characterization of each individual interface in these advanced, multifunctional polymer composites through experimental and theoretical proofs. From the current reviewed applications, the hybridizing of CNTs with natural fiber has potential use in the automotive, aerospace, marine, and sporting goods industries.

## Figures and Tables

**Table 1:** The advantages and limitations on some of the techniques used in the preparation of carbon nanotubes (CNTs). CVD: chemical vapor deposition.

Technique	Advantages	Limitations
Arc discharge	Arc discharge was based on a high voltage arc to produce plasma. Produced higher quality CNTs than thermally produced CVD materials.	Produce limited scale production of CNTs Produced by products such as soot and amorphous carbon and needs to be further purified. Produce random arrangement of CNTs.
Laser ablation	Produce high-quality CNTs in a large amount. Laser ablation utilizes a laser to add radiation energy to the synthesis reaction.	High-energy consumption Adhering impurities, thus requiring further purification.
CVD	Produce large scale of CNT production and vertical alignment of CNTs. Little impurities compared to arch discharge and laser ablation. Better control of CNT growth. Simple technique, consumes less energy, and is versatile.	Higher in CNT relative defectiveness. Complex process, difficulty in process controlling. Production of toxic and corrosive gases.

**Table 2:** Summary of advantages and limitations of fabrication techniques for CNT-reinforced polymer composites.

Technique	Advantages	Limitations	Reference
Melt mixing	Able to use for large scales industrial applications Available for all thermoplastic materials	Mixing processes able to damage the length of nanotubes	[116,117]
Solution mixing	Produces tougher material Low electrical percolation threshold Homogeneous dispersion of CNTs	Limited to polymers that are soluble in solvents More brittle samples Non-uniform and inferior properties Low surface density of CNTs Limited applications	[37,86,118,119,120]
Sonication	Enhancing the dispersion of CNTs Improves the mechanical properties	Breakage of CNTs causes the problem of mixing-induced fracture High-power and long sonication time reduce the aspect ratio of CNT Deterioration in properties	[90,94,116,118,121,122]
Resin transfer molding	Cost-effective High production rate Ideal for the production of complex shapes dissipation. Homogenous dispersion Unable to achieve the high loading and controllable orientation of CNTs Smooth surface on both sides Possibility for gelcoat on both sides Tolerance-stable work pieces	Effect of edge flow The smoothness and uniformity of the flow pattern can be disrupted Defects during the filling of the mold cavity Resin velocity may vary from point to point due to non-uniformity and rough fiber structure Formation of voids	[99]
Bucky paper resin infiltration	Simple way to create polymer nanocomposites with a high loading of CNTs Improves mechanical and electrical properties	High viscosity causes problems of dispersion Difficult to accomplish the complete impregnation of epoxy resin in bucky paper	[81,123,124,125]
Aligned CNT sheet processing	High volume fraction composites with desirable structural characteristics can be produced Effective in creating superior CNT sheets with high alignment and dense, high-performance structural composites	The mechanical properties of the composites may be degraded by the waviness and poor packing of CNTs in the sheets Hard to handle and to perform mechanical stretching	[102,107,126,127,128]
Shear Mixing	Exhibits better mechanical properties Able to separate the aggregates apart from each other Great technique for dispersion Effectively separates the CNTs without causing filament damage A three-roll mill -prepared epoxy composites have higher electrical conductivity	Unable to achieve a level of stress matching the density of binding energy Tubes will be broken for the full separation of long CNTs during the process A pulling effect (a tensile force) on the nanotube can be induced Dispersion techniques supplying high energy input may induce CNT fracture Shear mixing for a long time may lead to tube lengths being shortened, thus deteriorating composite properties	[32,90]
In-situ polymerization	Flexible, robust, and large-area membranes can been prepared with vertically aligned arrays of carbon nanotubes Produces pores will prevent nanotube alignment from blocking the pores and disturbing them Flexible, aligned CNT membranes with relatively high CNT density	Poor CNT alignment Increases viscosity along with progress	[110,117,129,130,131]

**Table 3:** Typical mechanical properties of CNT with other common structural materials. SWCNT: single-walled carbon nanotube; MWCTN: multiwalled carbon nanotube.

Type of CNT	Young’s Modulus (TPa)	Tensile Strength (GPa)
SWCNT	0.65–5.5	126
MWCNT	0.2–1.0	>63 (300)
Stainless steel	0.186–0.214	0.38–1.55
Kevlar	0.06–0.18	3.6–3.8
Diamond	1.22	>60 (225)
Aluminum	71	0.65
Glass Fibers	72	3
Carbon Fibers	300	3
Silicon Carbide Fibers	450	10

**Table 4:** Mechanical, electrical, and thermal properties of pure polyimide and a CNT/polyimide composite.

Sample	Tensile Strength (MPa)	Elastic Modulus (GPa)	Thermal Conductivity (W/mK^−1^)	Electrical Conductivity (Scm^−1^)
Pristine polyimide	227.70	4.04	0.027	10^−16^
CNT/polyimide	680	53.73	18.4	183.3

**Table 5:** Young’s modulus of polymethyl methacrylate (PMMA) reinforced with (5, 5) CNTs.

Aspect Ratio of CNT (L/d)	Young’s Modulus (MPa)
7.23	3.90
14.21	4.73
22.01	6.85
∞	46.73

**Table 6:** Tensile properties of polyimide/SWCNT composites.

SWCNT Loading (wt.%)	Tensile Strength (MPa)	Tensile Modulus (GPa)	Elongation (%)	Toughness (mJ/mm^3^)
0	74	2.2	175	123
0.1	86	2.6	125	100
0.3	94	2.8	110	92
1.0	100	3.2	20	6

**Table 7:** Summary of the mechanical properties of CNT–polymer composites from 2015 to 2020.

Type of CNT	Filler Content (%)	Matrix	Fabrication Technique	Tensile Strength (MPa)	Tensile Modulus (GPa)	Reference
Thermoplastic polymer						
MWCNTs	2 wt.%	PC	Grafting extrusion	61 (26% increase)	1.45	[159]
MWCNTs	8 wt.%	HDPE	Compression molding and blown film extrusion	18 (34% decrease)	2.3	[160]
MWCNTs	15 wt.%	PP	Melt mixing and extrusion	47 (38% increase)	0.37	[161]
Cu MWCNTs	2 wt.%	PLA/ESO	Mechanical stirring and sonication	0.8 (54% increase)	0.97 (33% increase)	[162]
MWCNTs	1.5 wt.%	TPU	Sonication and stirring	63 (40% increase)	0.095 (280% increase)	[163]
MWCNTs	10 wt.%	UHMWPE	Solution mixing and sintering	22 (37% increase)	0.25 (20% increase)	[164]
Amide MWCNTs	0.5 wt.%	WBPU	Sonication and stirring	12 (20% increase)	0.07 (10% increase)	[165]
Methanol MWCNTs	35 wt.%	TPU-acetone	Sonication	41 (20% increase)	1.27 (950% increase)	[165]
Iron (III) acetylacetonate MWCNTs	1.5 wt.%	TPU	Sonication and stirring	14 (100% increase)		[166]
MWCNTs	5 wt.%	PP	Grinding and injection molding	36	1.8	[167]
SWCNTs	1 wt.%	PS	Ultrasonication	12.7 (12% increase)	0.01 (11% decrease)	[168]
Acid MWCNT	5 wt.%	PMMA	Solution mixing	30 (200% increase)	1.3 (188% increase)	[169]
MWCNTs	1 wt.%	PP	Ultrasonication and hot-pressing	25	2	[170]
Thermosetting polymer						
ZnO MWCNTs	1.7 wt.%	Epoxy	Sonication	61 (20% increase)	3.6 (51% increase)	[171]
MWCNTs	56 wt.%	Epoxy	CVD, rolling, and hot-pressing		15.5	[148]
MWCNTs	1 wt.%	Epoxy	Ultrasonication	125 (160% increase)	-	[145]
Gelatin MWCNTs	0.5 wt.%	Epoxy	Mechanical mixing and sonication	98 (16% increase)	2.91 (18% increase)	[146]
MWCNTs	3 wt.%	Epoxy	Ultrasonication and sonication	31.42 (192% increase)	-	[172]
MWCNTs	3 wt.%	Epoxy	Ultrasonication and sonication	339.90	-	[172]
MWCNTs	1 wt.%	Epoxy	Ultrasonication and sonication	105% increase	-	[173]
MWCNTs	3 wt.%	Epoxy	Ultrasonication and sonication	52.225 (65% increase)	-	[174]
MWCNTs	3 wt.%	Epoxy	Ultrasonication	230.13 (70.6% increase)	-	[175]
MWCNTs	3 wt.%	Epoxy	Ultrasonication	24.83 (127% increase)	-	[175]
CNTs	5 wt.%	Epoxy	Extrusion and powder impregnation	81 (30% increase)	-	[77]
TA-PEI/MWCNTs	0.4 wt.%	Epoxy	Ultrasonication	80.83 (148% increase)	-	[77]
Straight CNTs	0.1 wt.%	Epoxy	Sonication and magnetic stirring	72.91 (13.21% increase)	25 (25.86% increase)	[175]
Helical CNTs	0.05 wt.%	Epoxy	Sonication and magnetic stirring	72.75 MPa (12.96% increase)	23.96 (25.24% increase)	[175]
MWCNTs	7.5–16 wt.%	Epoxy	Epoxidation and chemical treatment	203 MPa (50% increase)	8.4 (144% increase)	[77]
CNT fiber	0.1–2 wt.%	Epoxy	Direct spinning	2.1 N/tex (313% increase)	-	[100]
Pyrogallol MWCNT	1.5 wt.%	Epoxy	Mechanical mixing	76 (24% increase)	-	[144]
MWCNTs	0.4 wt.%	Epoxy	Mechanical mixing and hot pressing	42 (2% decrease)	2 (2% decrease)	[147]

Abbreviations: epoxidized soybean oil (ESO), high density polyethylene (HDPE), polycarbonate (PC), polyethylene (PE), polylactic acid (PLA), polyethyleneimine (PEI), polypropylene (PP), tannic acid (TA), thermoplastic polyurethane (TPU), ultra high molecular weight polyethylene (UHMWPE), waterborne polyurethane (WBPU).

**Table 8:** Transmissions in the thermal properties of various CNT–polymer composite materials based on their application.

Type of Polymer	Type of CNT	CNT Content (wt.%)	Improvement Properties	Potential Application	Reference
-	SWCNT	100 wt.%	Up to 3500 W/m·K	Heat sinks, connectors, batteries, light-emitting diode devices, automotive electronic control units, printed circuit boards, electronic assembly, and packaging	[177]
Polyacrylate composites	MWCNT	50–80 wt.%	˄ ~0.50 to 1.67 W/m K	Aerospace and aeronautics material	[183]
PLA	MWCNT	0.25–2.5 wt.%	27.5 mW·m^−1^K^−1^	High performance thermal insulator	[184]
Poly-dimethyl siloxane	MWCNT	~2 wt.%	˄ ~1.5 W/m·K	Thermal insulator/high thermal conductivity polymer composites	[185]
Poly-dimethyl siloxane/Si	MWCNT	~30%	˄ ~10% of 3000 W/m·K ≈3300 W/m·K		
Polymer-based composite	CNTs	30%	1000–4000 W/m·K	Light-emitting diodes and thermal dissipation	[179]
Polystyrene	MWCNT	1%	~30.2 mW/m-K without using any insulation gas	Super-thermal insulation properties	[186]
Poly (vinylidene fluoride)	MWCNT/graphene(1:1)	10% of (s-MWCNTs)/graphene (GE)	˄ ~711.1% of 0.19 W/m·K ≈135.11 W/m·K	Heat exchanger	[187]
PC	MWCNT	2%	˄ ~1.27 W/m·K	High performance thermal insulator	[188]
Resol-type phenolic resin	MWCNT	up to 1%	˄ ~35 °C of maximum degradation temperature	Strong network char layer without any cracks or opening	[189]
Carbon prepreg (IM7) composite	SWCNT	30%	˄ 30% heat capacity	Pipes and heat exchangers, replacer material for heavy-lift rocket under the Space Launch System (SLS), and promising high performance material for future space vehicles.	[190]
			˄ 30% thermal diffusivity		
			˄ ~120–150% thermal conductivity		
Epoxy	MWCNT	0.3 wt.%	˄ 35–42% of critical buckling temperature	Coefficient of thermal expansion (CTE) and thermal buckling of epoxy-based composites	[191]
Epoxy	SWCNT	1 wt.%	˄ ~125% W/m·K (method raw laser-oven)	High performance thermal insulator	[90]
		3 wt.%	˄ ~30% W/m·K		

**Table 9:** Electrical conductance of CNTs and polymers with potential applications.

Polymer	Types of CNT	CNT Content (wt.%)	Maximum Conductivity, σ_max_ (Sm^−1^)	Potential Applications	Reference
**Thermoplastics polymer**					
PP	MWCNT	≤3.5	21.9	Conductive adhesive, coating, and resistor	[193]
PC	SWCNT MWCNT	≤3.0 ≤10.0	1.0 × 10^2^ 0.9	Lightning strike protector, shielding, and coating	[198]
PMMA	MWCNT	≤3.0	2.0 × 10^−4^	Electromagnetic interference shielding	[217]
Ethylene-1-octene	MWCNT	2.0	2.0 × 10^−5^	Sensor, shielding	[218]
Pyridinium salt polymer	SWCNT	50% (content)	1.6 × 10^4^	Thermoelectric materials	[219]
PP	MWCNT	3.2 (vol.%)	1.2 × 10^7^	Electromagnetic shielding, anti-static cover, and chemical sensing	[210]
PANI	f-MWCNT	≤8.0	28.6	Electromagnetic interference shielding	[196]
**Thermosetting polymer**					
Derived epoxy	Hydroxyl-functionalized SWCNT	≤5.0	10^1^	Metal replacement, sensor, and shielding	[200]
Polyamide	MWCNT	7	6	Shielding, conductive adhesive, and coating	[194]
Epoxy	MWCNT	0.73	2.5 × 10^−2^	Sensor and shielding	[5]
Carbon fiber-reinforced polymer	SWCNT	-	5.0 × 10^5^	Lightning strike protector, shielding, coating, and thermoelectric materials	[212]
PVDF	MWCNT	0.5	0.8	Conductive adhesive and coating	[197]
Vinyl ester	MWCNT	≤0.5	10^−1^	Electromagnetic shielding and sensor	[220]
PPy	MWCNT	≤0.5	8.05 × 10^2^	Solar cells and Li-ion battery	[216]

**Table 10:** Occupational exposure limit values (OELs) for nanomaterial handling.

Description	Benchmark Exposure Level
Fibrous, a high aspect ratio insoluble nanomaterials	0.01 fibers/mL
Any nanomaterial that is already classified in its molecular or in its larger particle form a as carcinogenic, mutagenic, reproductive, and sensitizing (CMRS) toxin	0.1 × OEL
Insoluble or poorly soluble nanomaterials not in the fibrous or CMRS categories	0.066 × OEL
Soluble nanomaterials not in the fibrous or CMRS categories	0.5 × OEL

**Table 11:** Recent progress of CNT–polymer composites.

Applications	Types of CNT	Polymers	References
Biomedical goods, space vehicles, and stations	SWCNT	Poly (4-methyl-1-pentene)	[254]
Biocatalytic films	SWCNT	PMMA	[255]
Actuators and sensors for biomedical Applications	MWCNT	Poly (vinyl alcohol) and poly(2-acrylamido-2-methyl-1-propane sulfonic acid)	[256]
Supercapacitor electrode materials	MWCNT	PPy, Poly-(3,4-ethylenedioxythiophene) and PANI	[257]
External body components of automotive, yarn fiber, conductive plastic, and hot melt adhesives	MWCNT	PE	[258]
Electronics, electrostatic discharge, and automotive and industrial goods	SWCNT and MWCNT	Polyamide	[6]
Wind turbine blade and flame retardant	SWCNT and MWCNT	PU	[259]

**Table 12:** Yield strength and Young’s modulus at different strain levels and CNT loading.

CNTs (wt. %)	σ_10%_ (MPa)	Young’s Modulus (MPa)	Yield Strength (MPa)
0	4	E_O_ = 118	1
1	8	236 (2 × E_O_)	3
4	10	456 (3.9 × E_O_)	6

## Data Availability

Not applicable.
